# Treatment‐Related Adverse Events and Health‐Related Quality of Life Associated With Immune Checkpoint Inhibitor Treatment for Mucosal Head and Neck Squamous Cell Carcinoma: A Scoping Review

**DOI:** 10.1002/hed.70306

**Published:** 2026-06-01

**Authors:** N. D. O'Donnell, E. C. Ward, L. R. Wishart, R. Perera, C. Barrett, K. A. Edmiston, E. Mitchell, A. Pigott, R. Pitt, K. Russell, B. A. Chan, R. Ladwa

**Affiliations:** ^1^ School of Health & Rehabilitation Sciences The University of Queensland St Lucia Queensland Australia; ^2^ Sunshine Coast University Hospital, Queensland Health Birtinya Queensland Australia; ^3^ Centre for Functioning and Health Research Metro South Health, Queensland Health Buranda Queensland Australia; ^4^ Office of the Chief Allied Health Practitioner Metro North Health, Queensland Health Herston Queensland Australia; ^5^ School of Medicine and Dentistry Griffith University Brisbane Queensland Australia; ^6^ Speech Pathology Department Austin Health Melbourne Victoria Australia; ^7^ Princess Alexandra Hospital, Queensland Health Buranda Queensland Australia; ^8^ School of Medicine The University of Queensland St Lucia Queensland Australia

**Keywords:** head and neck cancer, immune checkpoint inhibitor, immunotherapy, quality of life, scoping review, supportive care, symptoms

## Abstract

**Background:**

Supportive care needs of patients receiving immune checkpoint inhibitors (ICIs) for head and neck squamous cell carcinoma (HNSCC) are ill‐defined. Hence, known treatment‐related adverse events (TRAEs) and health‐related quality of life (HRQoL) associated with ICI treatment for HNSCC were examined to inform future supportive care services.

**Methods:**

Scoping review including 98 studies.

**Results:**

Moderate–severe TRAEs occurred at < 10% incidence, with most experiencing mild impacts. Dermatologic, endocrine, pulmonary, hepatic, gastrointestinal, and constitutional complaints were reported in > 40% of studies, with most symptoms occurring during treatment. ICI monotherapy had less reported toxicity than multimodality treatments. Adding ICIs to multimodality treatment creates a minimal increase in TRAEs. HRQoL scores were low, with little change during ICI treatment.

**Conclusions:**

ICIs for HNSCC are generally well tolerated; however, TRAEs are common and may carry long‐term functional consequences. Flexible supportive care pathways are needed to assist early detection and individualized management of functional impacts to optimize survivorship.

## 1 Introduction

The introduction of immune checkpoint inhibitors (ICIs) for head and neck squamous cell carcinoma (HNSCC) has been a significant advancement, with the potential to transform disease management. Treatment targeting the programmed cell death protein‐1/programmed death‐ligand 1 (PD‐1/PD‐L1) pathway is now being employed across a range of clinical contexts using varied protocols for the management of both cutaneous and mucosal HNSCC [1]. ICIs were approved as a first‐line treatment option for recurrent or metastatic (R/M) HNSCC in 2016 in the United States and in 2022 in Australia. Landmark trials such as KEYNOTE 048 [2] and CheckMate 141 [3, 4] demonstrated superior survival with the use of ICIs compared to traditional chemotherapy‐based regimens for R/M HNSCC. Building on these findings, a growing number of clinical trials are now exploring the application of ICI treatment for the curative management of locally advanced (LA) HNSCC, where the addition of neoadjuvant and adjuvant pembrolizumab to standard of care has significantly improved event‐free survival among participants with LA resectable disease [5]. These early results have the potential to substantially alter future management of LA HNSCC.

With the integration of ICI in the first line palliative setting for both mucosal and cutaneous HNSCC, understanding the nature of ICI specific treatment‐related adverse events (TRAEs) and associated impacts for patients is integral to understanding both treatment tolerability and informing the early and ongoing supportive care needs into survivorship. Systematic reviews of ICIs involving a range of cancer populations have provided broad insights into TRAEs associated with ICI therapy. Typically, treatment with ICIs is regarded as “less toxic” than other systemic therapies [6, 7, 8, 9], and this is reported to extend to the HNSCC population [10]. Evidence from a meta‐analysis by Chen et al. [9] evaluating the efficacy and safety of ICI monotherapy compared to standard of care for R/M HNSCC found that the incidence of TRAEs was lower for ICI therapy than for chemotherapy and/or targeted agents. However, the clinical context of HNSCC is complex. Many patients have undergone intensive multimodal treatment resulting in substantial preexisting functional impairment and long‐term toxicities. Against this backdrop, even lower‐grade or lower‐incidence TRAEs may carry amplified consequences for daily functioning and HRQoL.

Despite the favorable toxicity profile of ICIs compared to other treatment modalities in HNSCC, TRAEs are still common. A recent systematic review by Dang et al. [11] exploring the overall safety and tolerability of ICIs for R/M HNSCC reported a 62.07% incidence of any‐grade TRAEs (13.82% incidence of severe Grade 3–4 TRAEs). These TRAEs encompass a diverse range of symptoms, with immune‐related adverse events (irAEs), a subset of TRAEs, resulting from immune system overactivation. TRAEs can occur either during or post treatment and vary widely in organ system involvement and severity [9, 10, 12]. Some of the most commonly reported TRAEs include rash, itching, and fatigue, though more severe and potentially life‐threatening side effects including pneumonitis, colitis, and neurological disorders can also occur [13, 14, 15].

Understanding the patient experience of ICI therapy in HNSCC requires further attention not only to better understand survival outcomes but also how both disease‐related morbidity and TRAEs shape HRQoL. Although a systematic review of PROs and HRQoL in cancer populations receiving ICIs (not specific to HNSCC) suggest these therapies are generally well tolerated, the evidence base is limited by few studies and substantial heterogeneity [6]. As ICI use in HNSCC expands across both the R/M and LA HNSCC contexts [12, 16], the lack of comparable data on treatment impacts becomes increasingly salient. Unlike surgery, radiation, or chemoradiotherapy, where long‐established toxicity profiles have informed supportive care pathways and services [17, 18], the functional and HrQOL consequences of ICI treatment in HNSCC remain poorly understood. This gap is particularly important given that many patients commence ICI treatment with substantial morbidity from previous therapies, meaning new TRAEs may compound existing impairments. To address this, the primary aim of this review is to conduct a scoping review of the literature specifically focusing on the nature, severity, and duration of TRAEs experienced by HNSCC patients receiving ICI treatment. The secondary aim is to synthesize current evidence on associated HrQOL and functional impacts for this specific population.

## 2 Methods

A scoping review methodology was selected to allow for the mapping of diverse literature on an emerging topic. The current review was conducted in accordance with the Joanna Briggs Institute updated methodology for scoping reviews [19] and reported using the Preferred Reporting Items for Systematic Reviews and Meta‐Analyses—Scoping Review (PRISMA‐ScR) [20]. The databases searched included MEDLINE, Embase, CINAHL, and Scopus, covering works published between January 2014 and October 2025.

Preliminary searches of MEDLINE, the Cochrane Database of Systematic Reviews, JBI Evidence Synthesis, and Open Science Framework were conducted, and two relevant reviews were identified. These were a systematic review by Dang et al. [11] which examined the overall incidence of TRAEs for R/M HNSCC patients treated with ICIs and a meta‐analysis by Chen et al. [9] comparing TRAEs experienced by R/M HNSCC patients treated with ICIs and those treated with standard of care regimens. These studies did not address HRQoL in this population, and descriptive data on TRAEs beyond overall severity grade were not provided. No comparable studies exploring ICI treatment in the LA HNSCC population were identified.

### 2.1 Search Strategy

A three‐step search strategy was used. First, an initial limited search of MEDLINE (PubMed) and CINAHL was undertaken to identify example articles on the topic. Keywords and index terms from key articles were used to inform a full search strategy in consultation with a health sciences librarian. The search strategy, including all identified keywords and index terms, was adapted for each included database (see Appendix 1). The search combined an extensive list of terms for three concepts: head and neck cancer AND immune checkpoint inhibitor therapy AND symptoms (search strategies for each database are available in Supporting Information).

### 2.2 Selection Criteria

Articles were included if they related to studies in which: (1) participants were adults diagnosed with mucosal HNSCC (cutaneous SCC was excluded), (2) participants received immune checkpoint inhibitors targeting the PD1/PDL1 pathway, administered as monotherapy or in combination with other treatment modalities, and (3) data included reporting of TRAEs (including irAEs) and/or HRQoL, and/or other reporting of associated physical, psychological or emotional symptoms experienced during or following treatment. Studies combining ICIs with agents other than standard of care chemotherapy, cetuximab, or radiation were reviewed by a medical oncologist to guide decision‐making regarding inclusion. Studies combining ICI therapy with an experimental agent, such as a Phase I trial agent, were excluded. Studies that reported on multiple cancer types or anatomical sites were only included if adverse event data could be disaggregated specifically for patients with HNSCC. To specifically elucidate the TRAEs associated with ICI monotherapy, an a priori decision was made to extract toxicity data only from studies that included a monotherapy ICI cohort, thereby removing confounding factors associated with multimodality treatment. Publications not available in English, review articles, conference abstracts, editorials, commentaries, case studies, and any papers that did not provide primary data were also excluded.

### 2.3 Article Screening

References were imported to Covidence [21] for the screening and review process; with duplicate articles automatically removed by Covidence following import. Title, abstracts, and full text screening were conducted by two of the authors (N.D.O., R.P.). Any disagreements were resolved through discussion until consensus was reached. All potentially relevant articles were then retrieved in full. Data extraction of all selected articles was conducted by the first author using a standardized template, with 20% of studies checked by a second reviewer (R.P.). The reference lists of all included papers were screened for additional articles.

### 2.4 Data Synthesis and Analysis

Data were extracted from included papers by a single reviewer (N.D.O.) guided by the Covidence 2.0 extraction template. Extracted data included study characteristics (date of publication and data collection, publication type, country of origin of the study, phase of study, study design, primary study objective, patient population characteristics [palliative vs. curative; monotherapy vs. combined therapy], immunotherapy agent); details of symptom collection (tools used, type and severity of AE screened for); main findings (types of symptoms reported, timing/incidence/severity/functional impact of symptoms); and HRQoL tools and findings. Type, severity, and incidence of symptoms experienced by participants during treatment with ICI monotherapy were extracted from relevant papers. Overall incidence of TRAEs, timing of onset, and occurrence of treatment cessation based on toxicity were compiled for all studies where possible. Symptom‐level data from studies involving ICI agents used in combination with other modalities (e.g., ICI + chemotherapy) were not extracted due to challenges in attributing causality. However, comparative data on the overall functional impact and extent of TRAEs were included when direct comparisons were made within different arms of a trial design.

## 3 Results

The search strategy yielded 2522 records, of which 1941 remained after removal of duplicates (Figure 1). Title and abstract screening excluded 1617 records, leaving 320 articles for full‐text review. Among these 320 articles, four were reports from the same clinical trial; only papers presenting novel information relevant to the current review were retained to avoid duplication. In total, 101 papers, representing 98 unique studies, met eligibility criteria and were included in the review.

**FIGURE 1:**
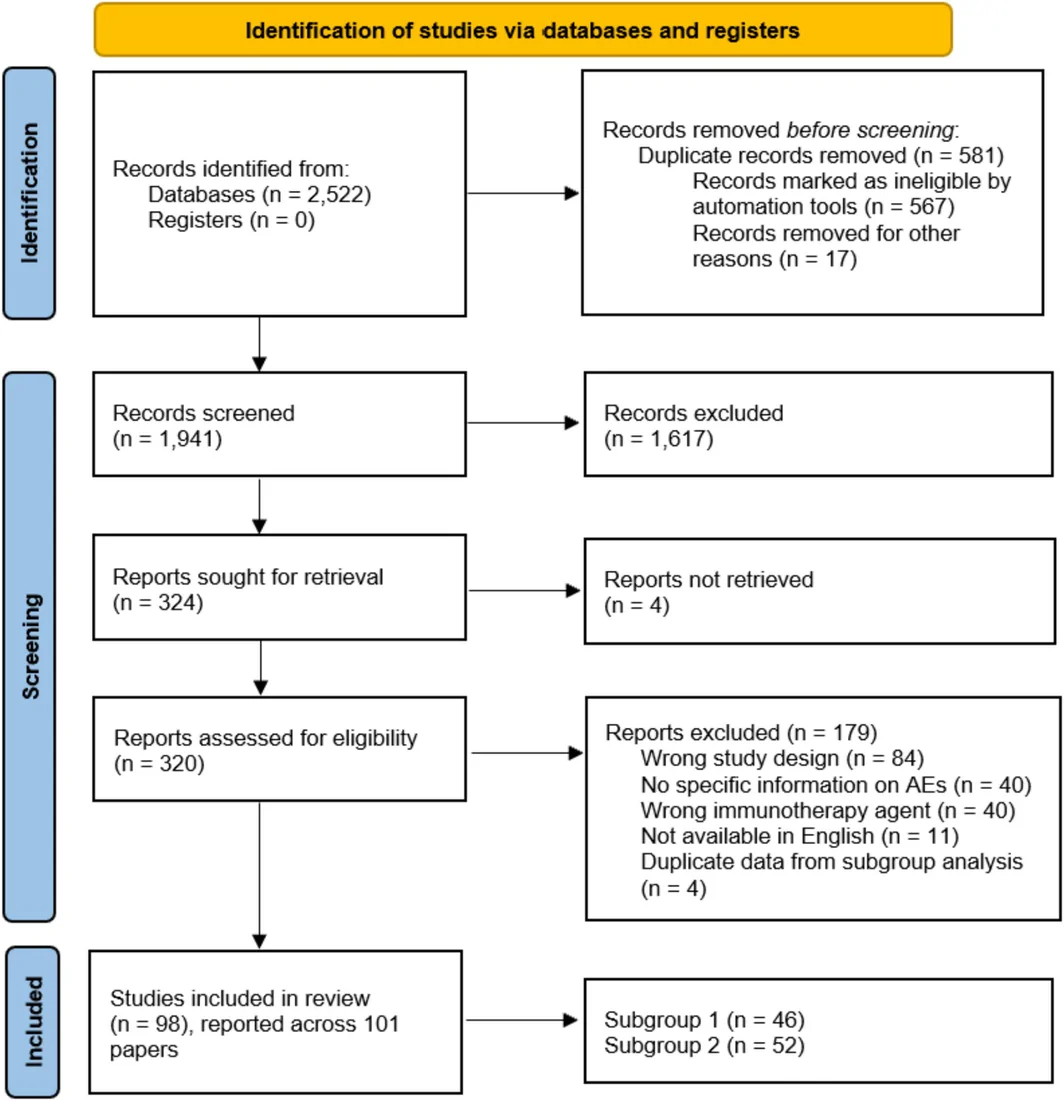
PRISMA diagram. Subgroup 1: ICIs used as monotherapy for HNSCC. Subgroup 2: ICIs used in combination with other therapies for HNSCC. [Color figure can be viewed at wileyonlinelibrary.com]

### 3.1 Study Characteristics

Across the 98 selected studies, the majority (*n* = 85) involved anti PD‐1 ICIs (nivolumab, cemiplimab, pembrolizumab, camrelizumab, toripalimab, and sintilimab), the most common of which being nivolumab (*n* = 44) and pembrolizumab (*n* = 39). Anti PD‐L1 agents (durvalumab, avelumab, and atezolizumab) were used in 13 studies. Most studies (*n* = 71) explored the efficacy of ICIs in palliative R/M HNSCC, with 27 studies conducted in curative‐intent LA HNSCC. Included studies were divided into two subgroups for analysis. Subgroup 1 (*n* = 46) included studies with TRAE data provided for participants receiving ICI monotherapy. Subgroup 2 (*n* = 52) included studies where ICIs were used in combination with other therapies, and no monotherapy cohort was reported on (Figure 1).

Of the 46 studies in Subgroup 1, 29 (63%) were retrospective cohort design, 15 (32%) were clinical trials, and 2 (4%) were prospective cohort design. Studies were completed in seven countries with the highest numbers conducted within Japan (19/46) and the United States (9/46). Ten studies were conducted across international cohorts. Most were single cohort studies, with only 11 (24%) involving a comparator arm. TRAE presence, frequency of incidence, severity, and timing, as well as HRQoL data were extracted.

Twenty‐four of the 52 studies in Subgroup 2 (47%) explored ICI combination therapy (ICI + systemic therapies or radiation therapy) in the palliative setting for R/M HNSCC (Table S1). These studies were conducted across nine countries, including eight from the United States and five from China, as well as several international cohorts. Study design included clinical trials (13/24), retrospective cohort studies (11/24), and one prospective cohort study. Fourteen (14/24) of the palliative intent studies combined ICIs with systemic therapy (chemotherapy and/or targeted therapy), 5/24 with radiation therapy, and 6/24 with both systemic therapy and radiation therapy.

The other 28/52 studies in Subgroup 2 included data on participants with LA HNSCC receiving ICI therapy as part of a curative treatment regimen (Table S2). Most of these studies (26/28) were clinical trials, with 2/28 retrospective cohort studies. These were conducted across four countries and international cohorts. For Subgroup 2, studies were only analyzed for TRAE data if they provided direct comparisons on TRAE incidence and severity between ICI‐containing combination regimens and comparator treatments with known AE profiles. This ensured that TRAE effects attributable to the addition of an ICI could be meaningfully assessed.

### 3.2 ICI Monotherapy for R/M HNSCC—Reported TRAEs

Research involving ICI monotherapy for R/M HNSCC totaled 49 papers relating to 46 studies. TRAEs were reported in 44/46 studies. However, the specific nature of TRAE reporting varied between the studies (Table 1), with 20 reporting all TRAEs (5 of these reporting TRAEs and irAEs of special interest separately), 24 reporting any‐grade irAEs only, and one reporting only Grade 3–4 irAEs [57]. Most studies (35/44) used the Common Terminology Criteria for Adverse Events (CTCAE) to report TRAEs (Table 1). Siu et al. [55] used the MD Anderson Symptom Inventory—Head and Neck module (MDASI‐HN [68]). Kirtane et al. [39] used patient‐reported measures to record TRAEs and functional impacts, including the Functional Assessment of Cancer Therapy—Immune Checkpoint Modulator (FACT ICM [69]) and the Functional Assessment of Cancer Therapy—Head and Neck (FACT HN [70]). Seven did not report the tool used, although in these studies TRAE descriptions were generally consistent with MedDRA terminology.

**TABLE 1:** ICI monotherapy for R/M HNSCC: *n* = 46 studies (Subgroup 1).

Study	Country	Design	Primary study objective(s)	Agent(s) used	*N* receiving monotherapy	Comparator arm(s) present	Adverse event reporting tool	Reporting style AEs	HRQoL reporting tool
Abbas et al. [22]	India	Retrospective cohort study	Evaluate efficacy and safety	Nivolumab	20	Nil	CTCAE	Any TRAE	None used
Arihara et al. [23]	Japan	Retrospective cohort study	Evaluate OS	Nivolumab	44	Nil	CTCAE	Any TRAE	None used
Bauml et al. [24]	United States	Non randomized phase 2 trial	Evaluate efficacy and safety	Pembrolizumab	171	Nil	CTCAE	TRAEs with incidence rate of ≥ 2%	None used
Burtness et al. [2]	International	Randomized phase 3 trial	Evaluate efficacy and safety	Pembrolizumab	300	ICI + chemo; SOC	CTCAE	TRAEs occurring in ≥ 15% of participants	None used
Cesaire et al. [25]	France	Retrospective cohort study	Evaluate impact of lymphopenia on efficacy	Nivolumab	100	Nil	Not specified	Any TRAE	None used
Chow et al. [26]	International	Non randomized phase 1 trial	Evaluate efficacy and safety	Pembrolizumab	132	Nil	CTCAE	Any TRAE	None used
Cohen et al. [27]	International	Randomized phase 3 trial	Evaluate efficacy and safety	Pembrolizumab	247	SOC	CTCAE	Any TRAE or irAEs of interest	None used
Colevas et al. [28]	France	Non randomized phase 1 trial	Evaluate efficacy and safety	Atezolizumab	32	Nil	CTCAE	Any TRAE	None used
Du et al. [29]	United States	Retrospective cohort study	Evaluate efficacy, safety, and demographics in a real‐world setting	Nivolumab	34	Nil	CTCAE	Any irAE	None used
Economopoulou et al. [30]	Greece	Retrospective cohort study	Evaluate the occurrence of irAEs and impact on survival	Nivolumab	89	Nil	CTCAE	Any irAE	None used
Ferris et al. [4]	International	Randomized phase 3 trial	Evaluate efficacy and safety	Nivolumab	240	SOC	CTCAE	Any TRAE with incidence of ≥ 5%	EORTC QLQ C30; EORTC QLQ HN‐35; ED‐5D‐3L
Foster et al. [31]	United States	Retrospective cohort study	Evaluate the association between development of irAEs and OS	Pembrolizumab Nivolumab Durvalumab Cemiplimab	108	Nil	CTCAE	Any irAE	None used
Gogate et al. [32]	International	Retrospective cohort study	Evaluate safety, efficacy, and HRQoL	Nivolumab	481	Nil	None used	—	EORTC QLQ C30; EORTC QLQ HN‐35; ED‐5D‐3L; others
Haas et al. [33]	Austria	Retrospective cohort study	Evaluate prognostic significance of baseline and on‐treatment thyroid dysfunction	Pembrolizumab	95	ICI + chemo	Not specified	Any irAE	None used
Harrington et al. [3]	International	Randomized phase 3 trial	Report effect of treatment on PROs	Nivolumab	240	Placebo	None used	—	EORTC QLQ C30; EORTC QLQ HN‐35
Harrington et al. [34]	International	Randomized phase 2 trial	Evaluate ORR	Nivolumab	82	PD‐1 + CTLA‐4	Other/not reported	Any TRAE	None used
Harrington et al. [35]	International	Secondary analysis of randomized phase 3 trial	Evaluate impact of treatment on HRQoL	Pembrolizumab	241	SOC	None used	—	EORTC QLQ C30; EORTC QLQ HN‐35; ED‐5D‐3L
Ishihara et al. [36]	Japan	Retrospective cohort	Evaluate association between AEs, treatment response, and prognosis	Pembrolizumab Nivolumab	92	Nil	CTCAE	Any irAE	None used
Iwasa et al. [37]	Japan	Retrospective cohort	Evaluate effectiveness and safety in real‐world setting	Nivolumab	46	Nil	CTCAE	Any irAE	None used
Jungbauer et al. [38]	Japan	Retrospective cohort	Identify risk factors for development of AEs	Pembrolizumab Nivolumab	35	Nil	Not specified	Any irAE	None used
Kirtane et al. [39]	United States	Prospective cohort	Report effect of treatment on PROs	Pembrolizumab Nivolumab	48	ICI + chemo	FACT‐ICM; FACT‐HN	Top 10 patient reported toxicities	FACT‐ICM; FACT‐HN
Kondo et al. [40]	Japan	Retrospective cohort	Evaluate efficacy and safety by age	Nivolumab	88	Nil	CTCAE	Any irAE	None used
Leddon et al. [41]	United States	Non randomized phase 2 trial	Evaluate efficacy and safety	Nivolumab	39	Nil	CTCAE	Any TRAE with incidence ≥ 5% (G1–2); any G3–4	None used
Matsuo et al. [42]	Japan	Retrospective cohort	Evaluate association between AEs, treatment response, and prognosis	Nivolumab	108	Nil	CTCAE	Any irAE	None used
Mehra et al. [43]	International	Secondary analysis of nonrandomized phase 1 trial	Evaluate efficacy and safety	Pembrolizumab	192	Nil	CTCAE	Any TRAE with incidence ≥ 2%	None used
Minohara et al. [44]	Japan	Retrospective cohort	Investigate the impact of clinico‐hematological factors on survival	Nivolumab	126	Nil	CTCAE	Any irAE	None used
Okada et al. [45]	Japan	Retrospective cohort	Evaluate efficacy and safety	Pembrolizumab	154	Nil	CTCAE	Any irAE	None used
Okamoto et al. [46]	Japan	Non randomized phase 2 trial	Evaluate efficacy, safety, and HRQoL	Nivolumab	22	Nil	CTCAE	Any irAE	EORTC QLQ C30; EORTC QLQ HN‐35
Otsuki et al. [47]	Japan	Retrospective cohort	Evaluate OS and PFS over 2 years	Nivolumab	88	Nil	CTCAE	Any irAE	None used
Otsuru et al. [48]	Japan	Retrospective cohort	Evaluate efficacy and safety	Nivolumab Pembrolizumab	53	ICI+ chemo; SOC	CTCAE	Any irAE	None used
Pareek et al. [49]	India	Retrospective cohort	Evaluate efficacy and safety	Nivolumab	12	Nil	Other/not reported	Any TRAE	None used
Rischin et al. [50]	International	Secondary analysis of randomized phase 3 trial	Evaluate impact of treatment on HRQoL	Pembrolizumab	301	ICI+ chemo	None used	—	EORTC QLQ C30; EORTC QLQ HN‐35; ED‐5D‐3L
Sano et al. [51]	Japan	Retrospective cohort	Evaluate effectiveness and safety in real‐world setting	Pembrolizumab	16	ICI + chemo	CTCAE	Any irAE	None used
Segal et al. [52]	United States	Non randomized phase 1/2 trial	Evaluate efficacy and safety	Durvalumab	62	Nil	CTCAE	Any TRAE with incidence ≥ 2% (specified AEs of special interest, defined as inflammatory or immune mediated)	None used
Seiwert et al. [53]	United States	Non randomized phase 1 trial	Evaluate efficacy and safety	Pembrolizumab	60	Nil	CTCAE	Any TRAE	None used
Simon et al. [54]	France	Retrospective cohort study	Assess the utilization of each treatment modality in a real‐ world setting	Pembrolizumab	56	ICI + chemo	CTCAE	Any TRAE with incidence ≥ 15% (G1‐2); and ≥ 5% (G3–4)	None used
Siu et al. [55]	United States	Randomized clinical trial	Evaluate efficacy and safety	Durvalumab	15	PD‐L1 + CTLA‐4	CTCAE MDASI‐HN	Any TRAE	None used
Tahara et al. [56]	Japan	Prospective cohort study	Evaluate efficacy and safety	Nivolumab	598	Nil	CTCAE	Any TRAE	None used
Thapa et al. [57]	Scotland	Retrospective cohort study	Evaluate effectiveness and safety in real‐world setting	Pembrolizumab	91	ICI + chemo	CTCAE	Any irAE ≥ G3	None used
Tsutomu et al. [58]	Japan	Retrospective cohort study	Evaluate prognostic and predictive markers of treatment	Nivolumab	29	Nil	Not specified	Any irAE	None used
Ueki et al. [59]	Japan	Retrospective cohort study	Evaluate prognostic factors of treatment	Nivolumab	42	Nil	CTCAE	Any irAE	None used
Ueki et al. [60]	Japan	Retrospective cohort study	Evaluate predictors of AEs	Nivolumab	62	Nil	CTCAE	Any irAE	None used
Vasiliadou et al. [61]	United Kingdom	Retrospective cohort study	Evaluate effectiveness and safety in real‐world setting	Nivolumab	123	Nil	Not specified	Any irAE	None used
Vasiliadou et al. [62]	United Kingdom	Retrospective cohort study	Evaluate effectiveness and safety in real‐world setting	Pembrolizumab	211	Nil	CTCAE	Any irAE	None used
Yamakawa et al. [63]	Japan	Retrospective cohort study	Evaluate efficacy and safety	Nivolumab	143	Nil	CTCAE	Any irAE	None used
Yasumatsu et al. [64]	Japan	Retrospective cohort study	Evaluate OR after treatment discontinuation	Nivolumab	14	Nil	CTCAE	Any irAE	None used
Yasumatsu et al. [65]	Japan	Retrospective cohort study	Evaluate efficacy and safety with long‐term data	Nivolumab	256	Nil	Not specified	Any irAE	None used
Yen et al. [66]	Asia	Randomized phase 3 trial	Evaluate efficacy and safety with long‐term data	Nivolumab	23	SOC	CTCAE	Any TRAE with incidence ≥ 10%	None used
Zandberg et al. [67]	International	Non randomized phase 2 trial	Evaluate efficacy and safety	Durvalumab	112	Nil	CTCAE	Any TRAE	EORTC QLQ‐C30; EORTC QLQ‐HN35

Abbreviations: Chemo = chemotherapy; CTCAE = Common Terminology Criteria for Adverse Events; G1 = Grade1; G2 = Grade 2; G3 = Grade 3; G4 = Grade 4; irAE = immune related adverse event; OS = overall survival; PFS = progression free survival; PROs = patient reported outcomes; SOC = standard of care; TRAE = treatment‐related adverse event.

The most commonly reported TRAEs included dermatologic symptoms (rash in 23/44 studies; pruritus in 14/44), endocrine disturbances (hypothyroidism in 28/44 of studies), pulmonary toxicities (pneumonitis in 18/44; interstitial lung disease in 15/44), hepatobiliary disorders (liver dysfunction in 14/44; hepatitis in 9/44), gastrointestinal symptoms (diarrhea in 23/44; colitis in 16/44 and nausea in 16/44), and fatigue in 21/44 studies. Reported incidence of TRAEs was noted to vary markedly between studies. For instance, Grade 1–2 (mild to moderate) fatigue affected as few as 1% of participants in some studies [48], while in others it was reported at 83% [49]. Similarly, Grade 1–2 (mild to moderate) diarrhea ranged from 2% [53] to 17% [49].

With regards to severity, most TRAEs were recorded as occurring at mild to moderate severity (Grade 1–2), with incidence rates of severe adverse events (Grade 3–4) generally occurring at rates well below 10% (Table 2). A notable exception was reported by Kirtane et al. [39], who found an incidence of severe headache in 23% of participants; however, this was recorded via patient‐reported measures. Treatment‐related toxicities resulting in discontinuation of ICI monotherapy were reported in 20 of the 44 studies. Rates of discontinuation were typically less than 10%, though this varied across studies from 0% [52] to 16.7% [47] (Figure 2).

**TABLE 2:** Monotherapy treatment‐related adverse event incidence.

Adverse event	Number of studies AE reported in % (*n* = 44)	G1–2^a^ incidence range (%)	G3–4^a^ incidence range (%)	All grade incidence range^b^ (%)
*General disorders and administration site conditions*				
Fatigue	48 (21)	1 [48]–83 [49]	< 1 [62]–7 [54]	10 [67]–58 [39]
Asthenia	20 (9)	17 [49]	< 1 [4, 27]–1 [2]	1 [30]–33 [39]
Pyrexia	14 (6)	1 [42]	< 1 [2]–2 [54]	5 [52]–13 [2]
Infusion‐related reaction	11 (5)	2 [44]	< 1 [27]–4 [44]	< 1 [45]–3 [27, 56]
Chills	7 (3)	5 [41]	—	2 [43]–6 [28]
Mucosal inflammation	7 (3)	—	< 1 [27]–1 [2]	1 [4]–4 [2, 27]
Facial swelling	5 (2)	2 [26]	1 [43]–2 [26]	3 [43]
Edema—general	5 (2)	5 [41]	1 [26]	5 [41]
Edema—laryngeal	2 (1)	—	1 [26]	—
Edema—facial	2 (1)	—	1 [26]	—
Xerostomia	2 (1)	1 [48]	—	—
*Psychiatric disorders*				
Anxiety	2 (1)	—	—	21 [39]
Insomnia	2 (1)	—	—	34 [39]
*Infections and infestations*				
Influenza‐type illness	5 (2)	5 [41]	—	5 [41]–9 [28]
Infection	5 (2)	—	1 [26]–5 [54]	7 [54]
Neck abscess	2 (1)	—	2 [53]	—
*Neoplasms benign, malignant and unspecified*				
Tumor pain	5 (2)	—	< 1 [67]	6 [28]
*Eye disorders*				
Optic neuritis	5 (2)	1 [42, 47]	—	1 [47]
Uveitis	5 (2)	—	< 1 [31]	< 1 [45]
Conjunctivitis	2 (1)	1 [25]	—	—
Chorioretinitis	2 (1)	—	—	1 [33]
Optic papillitis	2 (1)	1 [47]	—	1 [47]
*Nervous system disorders*				
Headache	7 (3)	5 [41]	—	3 [52]–23 [39]
Peripheral neuropathy	5 (2)	—	—	< 1 [4]–3 [24]
Dizziness	5 (2)	—	—	2 [24]–5 [52]
Encephalitis	5 (2)	—	1 [42]	—
Paresthesia	2 (1)	5 [41]		5 [41]
Problems with balance	2 (1)	—	—	33 [39]
Cerebellitis	2 (1)	—	2 [23]	2 [23]
Diplopia	2 (1)	—		5 [23]
Guillain‐Barre syndrome	2 (1)	—	< 1 [27]	1 [27]
Myasthenia gravis	2 (1)	—	2 [44]	
*Respiratory, thoracic and mediastinal disorders*				
Pneumonitis	41 (18)	2 [26]–9 [29]	< 1 [31, 47, 67]–10 [22]	< 1 [62]–14 [38]
Interstitial lung disease	35 (15)	2 [42, 48]–14 [46]	< 1 [63]–8 [44]	2 [37, 45]–18 [46]
Cough	11 (5)	5 [41]	—	3 [24, 52]–29 [39]
Respiratory distress	2 (1)	—	1 [26]	—
Bronchospasm	2 (1)	6 [51]	—	—
Dyspnea	2 (1)	—	1 [52]	3 [52]
*Metabolism and nutrition disorders*				
Appetite decrease/anorexia	23 (10)	1 [48]–7 [26]	1 [43]–4 [54]	5 [24, 67]–38 [39]
Weight loss	11 (5)	8 [41]	1 [2]	1 [4]–21 [39]
Diabetes	11 (5)	< 1 [45]–2 [47]	< 1 [56]–1 [26, 43]	< 1 [45, 56]–2 [47]
Hyperglycemia	9 (4)	1 [26]	1 [26, 42]	1 [62]
Hyponatremia	9 (4)	—	< 1 [61]–3 [53]	2 [24, 43]
Hypokalemia	7 (3)	—	< 1 [67]–2 [2]	5 [54]–8 [2]
Dehydration	5 (2)	—	< 1 [67]–1 [26]	—
Tumor lysis syndrome	2 (1)	—	—	6 [28]
Diabetic ketoacidosis	2 (1)	—	1 [26, 43]	—
Hypomagnesaemia	2 (1)	—	—	4 [2]
Hypophosphatemia	2 (1)	—	< 1 [67]	
*Hepatobiliary disorders*				
Liver dysfunction	6 (14)	1 [47]–9 [46]	1 [47]–14 [46]	< 1 [45]–27 [55]
Hepatitis	20 (9)	1 [62]	< 1 [27, 67]–3 [71]	1 [27, 33]–5 [71]
Cholangitis	2 (1)	1 [42]	—	—
*Gastrointestinal disorders*				
Diarrhea	52 (23)	2 [53]–17 [49]	< 1 [2]–10 [58]	1 [71]–15 [2]
Colitis	36 (16)	1 [60]	< 1 [31]–8 [49]	1 [27]–11 [37]
Nausea	36 (16)	5 [26, 41]–20 [55]	< 1 [62]–1 [26]	4 [54]–20 [55]
Oral mucositis/stomatitis	29 (13)	1 [26]–5 [22]	< 1 [27, 45]–3 [41]	1 [45, 48]–4 [66]
Vomiting	14 (6)	5 [22]–8 [49]	< 1 [2]	2 [43]–11 [2]
Abdominal pain	5 (2)	1 [26]	1 [26]	6 [28]
Pancreatitis	5 (2)	—	< 1 [45]	< 1 [45]–13 [55]
Dysphagia	2 (1)	1 [26]	1 [26]	—
Constipation	2 (1)	—	< 1 [2]	20 [2]
*Blood and lymphatic system disorders*				
Anemia	16 (7)	17 [49]	< 1 [27]–5 [2]	4 [24, 43]–27 [55]
Neutropenia	7 (3)	—	< 1 [2]–2 [54]	2 [2]–4 [54]
Thrombocytopenia	7 (3)	< 1 [61]	< 1 [2]	1 [33]–2 [2]
Lymphocytopenia	5 (2)	13 [55]	2 [53]	13 [55]
Immune thrombocytopenic purpura	2 (1)	—	1 [26]	—
*Investigations*				
ALT increase	9 (4)	—	< 1 [67]–3 [53]	3 [43]–4 [24]
AST increase	9 (4)	—	< 1 [67]–3 [53]	3 [43, 52]–6 [24]
Alkaline phosphatase increase	5 (2)	—	1 [24]	2 [24]–3 [52]
Bilirubin increase	5 (2)	—	< 1 [67]	3 [24]
Neutrophil count decreased	5 (2)	—	< 1 [27]	< 1 [2]–1 [27]
Platelet count decreased	5 (2)	—	—	1 [36]–3 [2]
White blood cell count decreased	2 (1)	—	—	1 [2]
Hypoalbuminemia	2 (1)	25 [49]	—	—
Lipase increase	2 (1)	7 [55]	13 [55]	20 [55]
*Musculoskeletal and connective tissue disorders*				
Arthritis	36 (16)	1 [47]–10 [41]	< 1 [31]–3 [41]	1 [33, 47, 48]–13 [41]
Myalgia	16 (7)	2 [53]–5 [41]	2 [53]	3 [24, 43]–13 [55]
Myositis	14 (6)	1 [42]	1 [42]–5 [23]	1 [33, 40]–5 [23]
*Endocrine disorders*				
Hypothyroid	64 (28)	3 [48]–13 [41]	< 1 [27]–7 [58]	2 [45]–18 [2, 46]
Hyperthyroid	16 (7)	< 1 [62]–5 [46]	—	2 [27]–6 [33]
Adrenal dysfunction/insufficiency	32 (14)	2 [62]–17 [49]	< 1 [56]–3 [47]	< 1 [63]–5 [23]
Thyroid dysfunction not defined	18 (8)	8 [47]–25 [54]	< 1 [63]	2 [43]–20 [55]
Hypophysitis	14 (6)		< 1 [45]–1 [42]	< 1 [45]–1 [71]
*Cardiac disorders*				
Myocarditis	5 (2)	—	< 1 [45]–1 [42]	< 1 [45]
Atrial fibrillation	5 (2)	—	1 [43]–2 [53]	—
Congestive heart failure	5 (2)	—	1 [43]–2 [53]	—
Cardiac disorder non specified	5 (2)	—	—	< 1 [56, 67]
*Skin and subcutaneous tissue disorders*				
Rash	52 (23)	10 [22]–20 [55]	< 1 [2]–2 [23]	4 [30]–20 [55]
Pruritus	32 (14)	12 [53]–19 [51]	2 [53]–3 [28]	2 [48]–22 [66]
Skin disorder not defined	27 (12)	6 [47]–14 [44]	< 1 [56, 64]–8 [44]	< 1 [56]–20 [54]
Dermatitis	14 (6)	9 [46]	—	3 [2, 58]–12 [59]
Dry skin	9 (4)	—	—	1 [48]–5 [43]
Lichenoid dermatitis	7 (3)	< 1 [61]	—	1 [33]–6 [30]
Alopecia	5 (2)	5 [22]	—	< 1 [27]
Erythema	5 (2)	—	—	4 [45]–5 [52]
Pemphigoid	2 (1)	—	—	< 1 [45]
*Renal and urinary disorders*				
Renal dysfunction	20 (9)	1 [47]–13 [55]	< 1 [56]–3 [67]	< 1 [56, 65]–13 [55]
Frequent urination	2 (1)	—	—	26 [39]

Abbreviations: AE, adverse event; ALT, alanine aminotransferase; AST, aspartate aminotransferase; CTCAE, Common Terminology Criteria for Adverse Events; G1–2, Grade 1–2; G3–4, Grade 3–4.

^a^ G1–2 and G3–4 incidence ranges are reported according to severity grading as defined by CTCAE or MedDRA criteria.

^b^ All‐grade incidence range reflects data reported as “all grades,” or where severity was not specified or was assessed using an alternative grading system.

**FIGURE 2:**
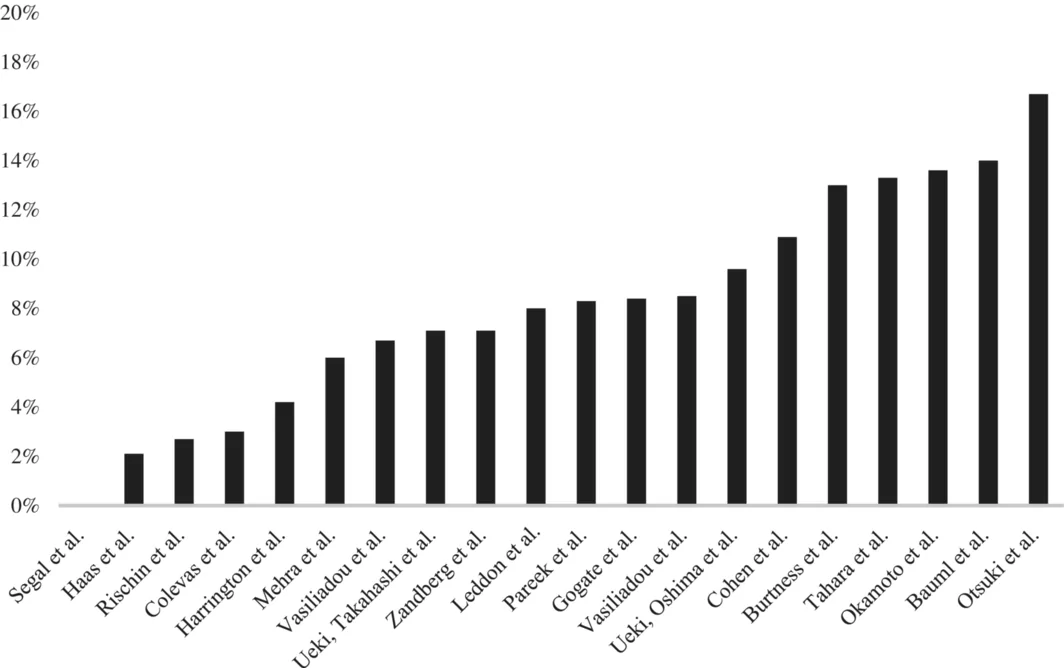
Monotherapy discontinuation data due to treatment‐related adverse event (monotherapy, R/M HNSCC studies).

Nine of the ICI monotherapy studies contained data regarding the timing of onset of TRAEs. Most toxicities were reported to occur within the first months of treatment with ICIs, with the median time of onset ranging from 4 to 14 weeks. Longer term (exceeding 2 years) follow‐up data was available from three studies [47, 65, 66]. These studies found only rare occurrences of new TRAEs occurring beyond the initial 12 months of ICI monotherapy. Otsuki et al. [47] reported that most TRAEs occurred within the first year of initial administration of nivolumab, with only 6/40 (15%) patients experiencing a TRAE after this point (data cut‐off occurred 24–36 months post commencement of nivolumab). Yasumatsu et al. [65] determined that 63% (39/62) of their patients experienced a TRAE during active treatment, with five (8.1%) patients developing a new TRAE post‐cessation of ICI treatment up to the end of the two‐year follow‐up period. Yen et al. [66] reported no new severe (Grade 3–4) TRAEs for CheckMate 141 participants during the interval between the data cut‐off of the initial safety and efficacy study by Kiyota et al. [72] (mean follow‐up 7.4 months) and their extended two‐year follow‐up.

Five studies [2, 39, 45, 54, 57] detailed the differences in adverse event profiles when ICI monotherapy was compared with combination regimens incorporating ICIs with another systemic agent (Table 3). All five studies demonstrated an increase in TRAEs with the addition of another systemic therapy to an ICI, with corresponding increases in discontinuation rates (where reported) due to treatment toxicity. For example, Burtness et al. [2] reported a 58% incidence of any‐grade TRAEs in the pembrolizumab monotherapy arm, compared with a 96% incidence in the pembrolizumab + chemotherapy arm. The discontinuation rates for the two arms were 13% and 18%, respectively. Similarly, Simon et al. [54] found a 73% incidence of TRAEs in the pembrolizumab monotherapy group, and a 95% incidence in the combined pembrolizumab + chemotherapy arm (this study did not report discontinuation data).

**TABLE 3:** Comparison of ICI monotherapy with ICI combined therapy TRAE incidence and discontinuation rates.

Study	Design	Treatment intent	Treatment arms	TRAE incidence	Discontinuation due to adverse events
Burtness et al. [2]	Randomized phase III trial	Palliative	Pembrolizumab	58% (≥ G3 17%)	13%
			Pembrolizumab + chemotherapy	96% (≥ G3 72%)	18%
			Cetuximab + chemotherapy	97% (≥ G3 69%)	16%
Kirtane et al. [39]	Prospective cohort study	Palliative	Pembrolizumab or nivolumab	No difference in severity of symptoms at baseline or up to 3 months between groups. Increase in severity of symptoms after 18–20‐week review	No discontinuation data
			Pembrolizumab/Nivolumab + Cetuximab		
Okada et al. [45]	Retrospective cohort study	Palliative	Pembrolizumab	17%	No discontinuation data
			Pembrolizumab + chemotherapy	22%	
Thapa et al. [57]	Retrospective cohort study	Palliative	Pembrolizumab	≥ G3 7.9%	0%
			Pembrolizumab + chemotherapy	≥ G3 13.3%	33%
Simon et al. [54]	Retrospective cohort study	Palliative	Pembrolizumab	73% (≥ G3 20%)	No discontinuation data
			Pembrolizumab + chemotherapy	95% (≥ G3 56%)	

Abbreviation: G3 = Grade 3.

### 3.3 ICI Monotherapy for R/M HNSCC—Health‐Related Quality of Life

Eight of the 46 ICI monotherapy studies reported data on HRQoL [3, 4, 32, 35, 39, 46, 50, 67] (Table 1). Tools to measure HRQoL frequently included the European Organization for Research and Treatment of Cancer (EORTC) Core Questionnaire (EORTC QLQ–C30), the head and neck specific module (EORTC QLQ–HN35), and the European Quality of Life–5 Dimensions (EQ‐5D‐3L) tool. Kirtane et al. [39] used the FACT–ICM and FACT–HN to assess both the presence of TRAEs as well as the functional impact on quality of life. In all included studies, active treatment with an ICI was not associated with a change in HRQoL, with scores remaining stable during an initial period of treatment and then declining in line with disease progression.

Four of these papers presented comparative HRQoL data between ICI monotherapy and chemotherapy arms [3, 4, 35, 50], and three of the four revealed better HRQoL during treatment for patients receiving ICI monotherapy. For example, in KEYNOTE‐040 [35], patients treated with pembrolizumab who remained on treatment at Week 15 had stable EORTC QLQ‐C30 global health status scores, whereas patients treated with chemotherapy experienced a clinically meaningful decline. Similarly, in the CheckMate 141 study [3], patients treated with nivolumab exhibited stable HRQoL scores from baseline to Weeks 9 and 15, whereas the chemotherapy group exhibited deterioration. However, in the KEYNOTE‐048 study [50], no significant differences in HRQoL scores between the pembrolizumab monotherapy treatment arm and the chemotherapy with cetuximab treatment arm were reported.

In addition to the small number of studies, it was also noted that HRQoL data were poorly collected in the long term due to patient death or increase in symptom burden. For example, in the study by Harrington et al. [35] there was a sharp decline in completion rates for the EORTC‐QLQ 30 from 95.5% completion at baseline to 48.1% after Week 15, with similar rates for the H&N‐35 and EQ‐5D measures. A similar pattern of reduced completion rates as a factor of time occurred across all studies looking at HRQoL in this population, with analysis of follow‐up data limited to between 8 [32] and 16 weeks [46].

### 3.4 ICI Combination Therapies for HNSCC

Fifty‐three papers (Subgroup 2) reported TRAE data for trials where ICI treatment was used in combination with other treatment modalities for R/M and LA HNSCC. Therapeutic combinations in the R/M HNSCC studies included ICI + radiation therapy (*n* = 5), ICI + chemotherapy (*n* = 10), ICI + cetuximab (*n* = 5), or tri/quad‐modality (ICI +/− surgery +/− radiation therapy +/− systemic therapy) (*n* = 6) (Table S1). When ICI therapy was used as a combination therapy in the LA HNSCC setting, the majority (*n* = 23) employed ICI in combination with tri/quad‐modality treatment; with ICI + radiation therapy (*n* = 2), ICI + surgery (*n* = 2) and ICI + chemotherapy (*n* = 1) being less frequent protocols (Table S2).

Given the complexity of combined treatment regimens and known synergistic toxicity of multimodality treatments, only information describing the impact of adding ICI therapy to existing systemic or radiotherapy protocols on overall tolerability and TRAE incidence was collated for these studies. Across the studies, five reported either no increase [73, 74] or a minimal increase [75, 76, 77] in TRAEs when an ICI was added to another systemic agent to intensify treatment. In KEYNOTE‐412 [75], which compared pembrolizumab + chemoradiotherapy followed by pembrolizumab maintenance with a placebo + chemoradiotherapy control for LA HNSCC, Grade 3–4 TRAEs increased from 88% in the placebo arm to 92% in the pembrolizumab arm. HRQoL was not significantly different between arms. Patil et al. [76] similarly found that the addition of an ICI to a chemotherapy regimen resulted in only a small increase in Grade 3–4 TRAEs from 46.1% to 50% of a cohort of participants with R/M HNSCC. The IMvoke010 trial [77] assessed the survival benefit and safety of administering maintenance atezolizumab following multimodal definitive therapy for LA HNSCC, and found a slight increase in TRAEs between the treatment arm (95%) versus placebo arm (91.6%), noting that immune‐related‐ AEs were more frequent in the atezolizumab arm (51.5%) compared with the placebo arm (35.5%). Li et al. [73] compared induction chemotherapy alone with induction chemotherapy plus an ICI for LA HNSCC and found no increase in toxicity in the combined treatment group. One study looked at the addition of an ICI to a palliative hypofractionated radiation treatment regimen [74] and found no significant increase in Grade 3+ TRAEs (radiation with ICI vs. radiation alone: 17.5% vs. 30.0%, *p* = 0.622), although comparative data for mild to moderate TRAEs was not available.

Within these combined treatment strategies, two studies compared the impact of substituting an ICI in place of another systemic agent. For example, in the GORTEC 2015‐01 PembroRad trial [78], radiation therapy with pembrolizumab was better tolerated than radiation therapy with cetuximab, with a significant reduction in Grade 3–4 TRAEs in the pembrolizumab arm (74%) compared with the cetuximab arm (92%, *p* = 0.006). The GORTEC 2017‐01 REACH trial [79] compared avelumab plus cetuximab with radiation against standard of care regimens, including cisplatin with radiation and cetuximab with radiation, and found no significant differences in Grade 3–4 TRAE rates across treatment arms.

## 4 Discussion

The purpose of this scoping review was to consolidate existing evidence on the TRAEs and impacts to HRQoL experienced by patients with HNSCC undergoing ICI treatment. Findings from the current review indicate that the rate of Grade 3 and above AEs was low, and the rate of discontinuation of ICI treatment attributed to treatment toxicity was also low. Overall, ICI treatment is well tolerated in this complex population. Despite this, a large proportion of patients experienced diverse Grade 1–2 TRAEs that may warrant enhanced supportive care. While ICI treatment in isolation does not appear to lead to a substantial HRQoL reduction, key gaps remain in understanding functional impacts and defining supportive care needs.

This review is the first to synthesize the reported TRAEs in ICI treatment for HNSCC. However, interpretation of the available evidence is limited by substantial inconsistency in AE reporting. Some studies reported all TRAEs; others focused only on selected irAEs, and several limited reporting to events above certain severity or frequency thresholds. This variability makes it difficult to draw conclusions about the risk, severity, and prevalence of specific AEs. Skin toxicities, thyroid dysfunction, pneumonitis, colitis, and liver dysfunction were among the most frequently reported, and these are broadly consistent with those experienced across other cancer types receiving ICI treatment [80]. Consequently, the available data offer only a partial understanding of the patient toxicity experience of receiving ICI therapy for HNSCC.

Regarding severity of TRAEs, studies in this review support that ICI monotherapy for HNSCC is typically associated with low overall toxicity, with relatively few patients reporting severe AEs compared with other palliative systemic therapies. This finding is consistent with the toxicity profile reported when ICIs have been used in the management of other cancer types [81, 82]. Equally, in most HNSCC studies in this review, discontinuation rates attributed to toxicities occurred at rates less than 10%. However, variability was observed, with 5 studies reporting higher discontinuation rates of between 13% and 16.7%, approaching the findings of the EXTREME trial [83], which reported discontinuation rates of approximately 20% for patients treated with chemotherapy with/without cetuximab for R/M HNSCC. This suggests that although ICI‐related TRAEs are typically mild, the cumulative effects of mild symptoms can impact treatment tolerability for some patients to a similar extent as other systemic agents. Further clarity is needed about the patterns of toxicities that lead to discontinuation of ICI monotherapy and what, if any, patient characteristics such as frailty or the presence of multiple comorbidities may also influence this.

Although the reported TRAEs were typically classified as mild, it is important to note that most of the existing studies obtained their symptom data from clinician‐reported outcome measures, typically using the CTCAE. PROMs were infrequently used to collect TRAEs. Considering the known differences in symptom reporting when clinician‐rated versus patient‐rated tools are used, there is potential for understated prevalence and/or missed functional impact reporting of TRAEs in studies to date. Atkinson et al. [84] in their systematic review found a poor to moderate association between CTCAE and PROMs ratings, with toxicities frequently either going undetected with CTCAE ratings or being reported as less severe than by PROMs. Indeed, in the study by Kirtane et al. [39] which was the only study that explored TRAEs using patient‐reported tools, 34% of patients reported experiencing moderate to severe insomnia and 21% experiencing moderate to severe anxiety, which are not TRAEs identified in any other studies in this review. They also found a higher incidence of fatigue and headache when compared with other studies that used only CTCAE. Hence, it is recognized that future research into ICI TRAEs needs to include PROMs to help to understand more fully the extent of symptom burden and functional impact of treatment [85]. Tools such as the PRO‐CTCAE, or the MDASI‐Immunotherapy module which has been specifically validated in the immunotherapy setting, may be particularly salient to capture the unique symptom burden of the TRAEs associated with ICIs. Incorporating such tools into future trials will help ensure a more comprehensive understanding of irAEs and functional impacts beyond clinician‐reported toxicity.

Data regarding the timing of emergence of TRAEs suggests that most emerge early (median of 4–14 weeks) post commencement of ICI treatment. New TRAEs following this period do occur, but with low frequency. This pattern is consistent with major immunotherapy toxicity guidelines, which note that most AEs emerge early in the course of treatment, typically within the first few weeks to months [86, 87]. Early monitoring for TRAEs during the initial period of treatment with an ICI will ensure appropriate supportive care can be provided in a timely manner.

Recognizing the diversity of symptoms and variability in severity and timing, consideration of resource‐efficient methods such as electronic PROM screening of TRAEs may be warranted for integration into clinical practice, to enable timely detection and management of early and late‐stage toxicities requiring medical management or supportive care. Electronic reporting platforms have demonstrated feasibility and effectiveness in improving symptom surveillance and patient outcomes in HNSCC patients receiving chemoradiotherapy [88, 89, 90]. Emerging evidence, including work by Lai‐Kwon and colleagues [91, 92] also supports the integration of such models into routine care pathways for immunotherapy. These approaches may enable proactive individualized intervention and reduce the risk of treatment discontinuation due to cumulative toxicity.

Although current evidence supports that ICI monotherapy is generally well‐tolerated, once ICIs are combined within multimodality treatment protocols, the rates of Grade 3–4 TRAEs increase sharply [2, 39, 45, 54, 57]. While this increase is largely driven by the known toxicity impacts of radiation and chemotherapy, studies have shown that a small but added increase in toxicities is directly associated with the addition of ICIs [75]. Hence, it is important to not lose sight of the fact that the overall impact of multimodality treatment including ICI therapy is significant for HNSCC, and overall treatment toxicity can be intensified with ICIs.

In contrast to TRAE reporting, the impact of ICI therapy on HRQoL remains less well explored with only 8% of studies in this review exploring impacts to quality of life or functional outcomes. Additionally, the available HRQoL data is restricted to a relatively brief period while on treatment, with reporting dropping off 2–4 months following commencement of an ICI. This attrition is often attributed to increasing symptom burden and reduced capacity to participate in research, suggesting that the HRQoL data captured may disproportionately reflect patients with better functional status. Further research on HRQoL beyond the initial weeks of ICI therapy is warranted. Despite this limitation in both the extent and the quality of data reported, the available evidence suggests that the use of ICIs in the palliative context does not significantly negatively impact HRQoL. Although HRQoL may not decline with ICI treatment, it is important to note that the baseline HRQoL reported in most studies is low compared to reference populations [93]. Patients presenting with R/M HNSCC are often heavily pre‐treated with multimodality treatment known to result in long‐term toxicities [94, 95], and are likely to also present with multiple comorbidities [96]. These patients are more likely to present with frailty [97] and associated reduced functional reserves. Within this context, the cumulative impact of even “mild,” but chronic low‐grade TRAEs can contribute to unacceptable treatment toxicity. This underscores the need to address wellbeing through effective multidisciplinary management of physical side effects and psychosocial needs to optimize HRQoL.

## 5 Limitations

The substantial heterogeneity in treatment regimens, patient populations, and symptom reporting practices, as well as the absence of standardized methods for capturing TRAE incidence limited the synthesis of results. Although broad search strategies were used, relevant studies may have been missed due to inconsistent terminology and variable indexing of ICI‐related toxicities. This review also focused only on studies involving mucosal HNSCC. Cutaneous squamous cell carcinoma (cSCC) of the head and neck region is a distinct clinical entity from HNSCC [98], and the vast majority of trials investigating cSCC include tumors outside the head/neck region. As such, cSCC was considered beyond the scope of this review, thus limiting generalizability of the study beyond mucosal HNSCC. However, given its shared anatomical location in many cases, cSCC patients may present overlapping functional and supportive care challenges. Including these patients in future symptom‐focused research will be essential to ensure supportive care strategies reflect the full spectrum of HNSCC presentations managed with ICI‐related toxicities.

## 6 Conclusion

Data from the ICI monotherapy studies support that symptoms experienced by HNSCC patients receiving ICI treatment are typically mild to moderate in severity, occur most frequently within the first few months of treatment, and affect all body systems with significant heterogeneity. When ICIs are used in combination with other multimodality treatments, the impact of the addition of an ICI is difficult to isolate, although some increase in toxicity is to be anticipated. HRQoL is often low at baseline and remains low in this population. Despite being classified as low grade, the cumulative effect of mild and/or chronic TRAEs can have meaningful consequences for HRQoL, underscoring the need for sensitive assessment and timely supportive care services. Issues with inconsistency in TRAE reporting and a paucity of data relating to the impacts of ICI treatment result in only a partial understanding of the functional consequences of ICI treatment for HNSCC. A greater understanding of TRAEs associated with ICI use in HNSCC management is needed to help guide best‐practice models for toxicity detection, functional impact monitoring, and optimal supportive care for this population.

## Funding

The authors have nothing to report.

## Disclosure

The authors have nothing to report.

## Ethics Statement

The authors have nothing to report.

## Conflicts of Interest

The authors declare no conflicts of interest.

## Supporting information


**Data S1:** Search strategies.

**Table S1:** Combined treatment in R/M HNSCC.
**Table S2:** Combined treatment in LA HNSCC.

## Data Availability

The data that support the findings of this study are available from the corresponding author upon reasonable request.

## Appendix 1 Search Strategy

1. Head and Neck Neoplasms/
2. (“head and neck cancer” or HNSCC or “head and neck squamous cell carcinoma”).ti,ab,kf
3. 1 or 2
4. Immunotherapy/
5. Antineoplastic Agents, Immunological/
6. Immune Checkpoint Inhibitors/
7. (Immunotherap* or “checkpoint inhibitor” or nivolumab or pembrolizumab or PD‐1 or PD‐L1).ti,ab,kf
8. 4 or 5 or 6 or 7
9. Signs and Symptoms/
10. Drug‐Related Side Effects and Adverse Reactions/
11. Symptom Burden/
12. Quality of Life/
13. Needs Assessment/
14. Psychological Distress/
15. (irAE or TRAE or “immune related adverse” or “immune‐related adverse” or symptom* or side‐effect* or “side effect*” or toxicit* or “treatment‐related adverse” or safety or “supportive care” or “unmet need” or “quality of life” or psycholog*).ti,ab,kf.
16. 9 or 10 or 11 or 12 or 13 or 14 or 15
17. 3 and 8 and 16
18. (last 10 years)

## References

[1] E. Yilmaz , N. Ismaila , J. E. Bauman , et al., “Immunotherapy and Biomarker Testing in Recurrent and Metastatic Head and Neck Cancers: ASCO Guideline,” Journal of Clinical Oncology 41, no. 5 (2023): 1132–1146. 10.1200/JCO.22.02328 36521102

[2] B. Burtness , K. J. Harrington , R. Greil , et al., “Pembrolizumab Alone or With Chemotherapy Versus Cetuximab With Chemotherapy for Recurrent or Metastatic Squamous Cell Carcinoma of the Head and Neck (KEYNOTE‐048): A Randomised, Open‐Label, Phase 3 Study,” Lancet 394, no. 10212 (2019): 1915–1928. 10.1016/S0140-6736(19)32591-7 31679945

[3] K. J. Harrington , R. L. Ferris , G. Blumenschein , et al., “Nivolumab Versus Standard, Single‐Agent Therapy of Investigator's Choice in Recurrent or Metastatic Squamous Cell Carcinoma of the Head and Neck (CheckMate 141): Health‐Related Quality‐Of‐Life Results From a Randomised, Phase 3 Trial,” Lancet Oncology 18, no. 8 (2017): 1104–1115. 10.1016/S1470-2045(17)30421-7 PMC6461049 28651929

[4] R. L. Ferris , G. Blumenschein , J. Fayette , et al., “Nivolumab for Recurrent Squamous‐Cell Carcinoma of the Head and Neck,” New England Journal of Medicine 375, no. 19 (2016): 1856–1867. 10.1056/NEJMoa1602252 PMC5564292 27718784

[5] R. Uppaluri , R. I. Haddad , Y. Tao , et al., “Neoadjuvant and Adjuvant Pembrolizumab in Locally Advanced Head and Neck Cancer,” New England Journal of Medicine 393, no. 1 (2025): 37–50. 10.1056/NEJMoa2415434 40532178

[6] E. Hall , S. Singhal , J. Dickerson , et al., “Patient‐Reported Outcomes for Cancer Patients Receiving Checkpoint Inhibitors: Opportunities for Palliative Care—A Systematic Review,” Journal of Pain and Symptom Management 58, no. 1 (2019): 137–156.e1. 10.1016/j.jpainsymman.2019.03.015 30905677

[7] J. M. Bauml , R. B. Cohen , and C. Aggarwal , “Immunotherapy for Head and Neck Cancer: Latest Developments and Clinical Potential,” Therapeutic Advances in Medical Oncology 8, no. 3 (2016): 168–175. 10.1177/1758834016631529 PMC4872249 27239235

[8] E. E. Cohen , K. J. Harrington , C. Le Tourneau , et al., “Pembrolizumab (Pembro) vs Standard of Care (SOC) for Recurrent or Metastatic Head and Neck Squamous Cell Carcinoma (R/M HNSCC): Phase 3 KEYNOTE‐040 Trial,” Annals of Oncology 28 (2017): v628.

[9] L. Chen , D.‐C. Mo , M. Hu , S.‐J. Zhao , Q.‐W. Yang , and Z.‐L. Huang , “PD‐1/PD‐L1 Inhibitor Monotherapy in Recurrent or Metastatic Squamous Cell Carcinoma of the Head and Neck: A Meta‐Analysis,” American Journal of Otolaryngology 43, no. 2 (2022): 103324. 10.1016/j.amjoto.2021.103324 34923281

[10] E. S. Sim , H. C. B. Nguyen , G. J. Hanna , and R. Uppaluri , “Current Progress and Future Directions of Immunotherapy in Head and Neck Squamous Cell Carcinoma: A Narrative Review,” JAMA Otolaryngology. Head & Neck Surgery 151, no. 5 (2025): 521–528. 10.1001/jamaoto.2024.5254 PMC12815376 40048196

[11] S. Dang , X. Li , H. Liu , S. Zhang , and W. Li , “Toxicity Profiles of Immune Checkpoint Inhibitors for Recurrent or Metastatic Head and Neck Squamous Cell Carcinoma: A Systematic Review and Meta‐Analysis,” Cancer Medicine 13, no. 7 (2024): e7119. 10.1002/cam4.7119 PMC10980932 38553943

[12] E. Cohen , R. Uppaluri , N. Lee , et al., “Neoadjuvant and Adjuvant Pembrolizumab (Pembro) Plus Standard of Care (SOC) in Patients (Pts) With Resectable Locally Advanced (LA) Head and Neck Squamous Cell Carcinoma (HNSCC): The Phase III KEYNOTE‐689 Study,” Annals of Oncology 30 (2019): ix106.

[13] T. Ouyang , Y. Cao , X. Kan , et al., “Treatment‐Related Serious Adverse Events of Immune Checkpoint Inhibitors in Clinical Trials: A Systematic Review,” Frontiers in Oncology 11 (2021): 621639. 10.3389/fonc.2021.621639 PMC8144509 34046338

[14] M. A. Postow , R. Sidlow , and M. D. Hellmann , “Immune‐Related Adverse Effects Associated With Immune Checkpoint Inhibitors,” New England Journal of Medicine 378 (2018): 158–168. 10.1056/NEJMra1703481 29320654

[15] B. Rapoport , R. Eeden , V. Sibaud , et al., “Supportive Care for Patients Undergoing Immunotherapy,” Supportive Care in Cancer 25, no. 10 (2017): 3017–3030. 10.1007/s00520-017-3802-9 28707167

[16] T. M. Wise‐Draper , S. Gulati , S. Palackdharry , et al., “Phase II Clinical Trial of Neoadjuvant and Adjuvant Pembrolizumab in Resectable Local‐Regionally Advanced Head and Neck Squamous Cell Carcinoma,” Clinical Cancer Research 28, no. 7 (2022): 1345–1352. 10.1158/1078-0432.CCR-21-3351 PMC8976828 35338369

[17] J. Hiatt , A. Young , T. Brown , M. Banks , and J. Bauer , “Improving Patient and Carer Access to Information and Support Through Head and Neck Cancer Treatment and Survivorship Using Experience‐Based Co‐Design,” Journal of Human Nutrition and Dietetics 36, no. 2 (2023): 443–452. 10.1111/jhn.13099 36218063

[18] W. K. W. So , C.‐L. Wong , K.‐C. Choi , et al., “A Mixed‐Methods Study of Unmet Supportive Care Needs Among Head and Neck Cancer Survivors,” Cancer Nursing 42, no. 1 (2019): 67–78. 10.1097/NCC.0000000000000542 28945632

[19] M. D. J. Peters , C. Marnie , A. C. Tricco , et al., “Updated Methodological Guidance for the Conduct of Scoping Reviews,” JBI Evidence Implementation 19, no. 1 (2021): 3–10. 10.1097/XEB.0000000000000277 33570328

[20] A. C. Tricco , E. Lillie , W. Zarin , et al., “PRISMA Extension for Scoping Reviews (PRISMA‐ScR): Checklist and Explanation,” Annals of Internal Medicine 169, no. 7 (2018): 467–473. 10.7326/M18-0850 30178033

[21] Veritas Health Innovation , Covidence Systematic Review Software (Melbourne, 2024), https://www.covidence.org/.

[22] W. Abbas , S. Gupta , V. Goel , et al., “Real‐World Experience of Immunotherapy From India in Recurrent Squamous Cell Carcinoma of Head and Neck Cancer,” South Asian Journal of Cancer 10, no. 2 (2021): 72–75. 10.1055/s-0041-1729443 PMC8460346 34568218

[23] Y. Arihara , K. Takada , K. Murase , et al., “Inflammation and Malnutrition as Markers of Poor Outcomes in Head and Neck Cancer Patients Treated With Nivolumab,” Acta Oto‐Laryngologica 143, no. 8 (2023): 714–720. 10.1080/00016489.2023.2240372 37537940

[24] J. Bauml , T. Y. Seiwert , D. G. Pfister , et al., “Pembrolizumab for Platinum‐ and Cetuximab‐Refractory Head and Neck Cancer: Results From a Single‐Arm, Phase II Study,” Journal of Clinical Oncology 35, no. 14 (2017): 1542–1549. 10.1200/JCO.2016.70.1524 PMC5946724 28328302

[25] M. Cesaire , A. Rambeau , F. Clatot , A. Johnson , N. Heutte , and J. Thariat , “Impact of Lymphopenia on Efficacy of Nivolumab in Head and Neck Cancer Patients,” European Archives of Oto‐Rhino‐Laryngology 280, no. 5 (2023): 2453–2461. 10.1007/s00405-022-07800-1 36564669

[26] L. Q. M. Chow , R. Haddad , S. Gupta , et al., “Antitumor Activity of Pembrolizumab in Biomarker‐Unselected Patients With Recurrent and/or Metastatic Head and Neck Squamous Cell Carcinoma: Results From the Phase Ib KEYNOTE‐012 Expansion Cohort,” Journal of Clinical Oncology 34, no. 32 (2016): 3838–3845. 10.1200/JCO.2016.68.1478 PMC6804896 27646946

[27] E. E. W. Cohen , D. Soulières , C. Le Tourneau , et al., “Pembrolizumab Versus Methotrexate, Docetaxel, or Cetuximab for Recurrent or Metastatic Head‐And‐Neck Squamous Cell Carcinoma (KEYNOTE‐040): A Randomised, Open‐Label, Phase 3 Study,” Lancet 393, no. 10167 (2019): 156–167. 10.1016/S0140-6736(18)31999-8 30509740

[28] A. D. Colevas , R. Bahleda , F. Braiteh , et al., “Safety and Clinical Activity of Atezolizumab in Head and Neck Cancer: Results From a Phase I Trial,” Annals of Oncology 29, no. 11 (2018): 2247–2253. 10.1093/annonc/mdy411 30219915

[29] Y. Du , R. Fu , J. T. Levinsky , et al., “Nivolumab for Recurrent or Metastatic Head and Neck Squamous Cell Carcinoma: A Retrospective Tertiary Centre's Real‐World Experience,” Current Oncology 30, no. 10 (2023): 8928–8935. 10.3390/curroncol30100645 PMC10604932 37887545

[30] P. Economopoulou , I. Kotsantis , G. Papaxoinis , et al., “Association of Autoimmunity With Survival in Patients With Recurrent/Metastatic Head and Neck Squamous Cell Carcinoma Treated With Nivolumab,” Oral Oncology 111 (2020): 105013. 10.1016/j.oraloncology.2020.105013 32977184

[31] C. C. Foster , M. A. Couey , S. E. Kochanny , et al., “Immune‐Related Adverse Events Are Associated With Improved Response, Progression‐Free Survival, and Overall Survival for Patients With Head and Neck Cancer Receiving Immune Checkpoint Inhibitors,” Cancer 127, no. 24 (2021): 4565–4573. 10.1002/cncr.33780 34547103

[32] A. Gogate , B. Bennett , Z. Poonja , et al., “Phase 4 Multinational Multicenter Retrospective and Prospective Real‐World Study of Nivolumab in Recurrent and Metastatic Squamous Cell Carcinoma of the Head and Neck,” Cancers (Basel) 15, no. 14 (2023): 3552. 10.3390/cancers15143552 PMC10377225 37509217

[33] M. Haas , A. Lein , T. Fuereder , et al., “Low Free Triiodothyronine and Immune‐Related Hyperthyroidism Are Associated With Overall and Progression‐Free Survival in Head and Neck Squamous Cell Carcinoma Treated With Pembrolizumab,” International Immunopharmacology 123 (2023): 110799. 10.1016/j.intimp.2023.110799 37598631

[34] K. J. Harrington , H. R. Kim , S. Salas , et al., “Lenvatinib With or Without Pembrolizumab Versus Chemotherapy for Treatment of Recurrent or Metastatic Head and Neck Squamous Cell Carcinoma That Progressed on Platinum Therapy and Immunotherapy: Phase 2 LEAP‐009,” Clinical Cancer Research 29, no. 18 (2023): PO‐003.

[35] K. J. Harrington , D. Soulières , C. Le Tourneau , et al., “Quality of Life With Pembrolizumab for Recurrent and/or Metastatic Head and Neck Squamous Cell Carcinoma: KEYNOTE‐040,” Journal of the National Cancer Institute 113, no. 2 (2021): 171–181. 10.1093/jnci/djaa063 PMC7850527 32407532

[36] H. Ishihara , Y. Asahi , M. Kuroki , et al., “Prognosis After Discontinued Treatment due to Immune‐Related Adverse Events in Recurrent and Metastatic Head and Neck Squamous Cell Carcinoma,” Head & Neck 48 (2025): 449–456. 10.1002/hed.70043 40937641

[37] Y.‐I. Iwasa , Y. Yokota , R. Kitoh , et al., “Factors Affecting Nivolumab Therapy Outcome in Patients With Head and Neck Cancer: A Single‐Center Analysis,” Oncology 100, no. 4 (2022): 203–211. 10.1159/000522035 35042219

[38] F. Jungbauer , A. Affolter , C. Brochhausen , et al., “Risk Factors for Immune‐Related Adverse Effects During CPI Therapy in Patients With Head and Neck Malignancies—A Single Center Study,” Frontiers in Oncology 14 (2024): 1287178. 10.3389/fonc.2024.1287178 PMC10899674 38420014

[39] K. Kirtane , A. I. Hoogland , X. Li , et al., “Patient‐Reported Outcomes in Immunotherapy for Head and Neck Cancer,” Head & Neck 45, no. 7 (2023): 1761–1771. 10.1002/hed.27388 37141438

[40] T. Kondo , I. Okamoto , H. Sato , et al., “Age‐Based Efficacy and Safety of Nivolumab for Recurrent or Metastatic Head and Neck Squamous Cell Carcinoma: A Multicenter Retrospective Study,” Asia‐Pacific Journal of Clinical Oncology 16, no. 6 (2020): 340–347. 10.1111/ajco.13374 32573033

[41] J. L. Leddon , S. Gulati , S. Haque , et al., “Phase II Trial of Adjuvant Nivolumab Following Salvage Resection in Patients With Recurrent Squamous Cell Carcinoma of the Head and Neck,” Clinical Cancer Research 28, no. 16 (2022): 3464–3472. 10.1158/1078-0432.CCR-21-4554 PMC9378458 35653116

[42] M. Matsuo , R. Yasumatsu , M. Masuda , et al., “Relationship Between Immune‐Related Adverse Events and the Long‐Term Outcomes in Recurrent/Metastatic Head and Neck Squamous Cell Carcinoma Treated With Nivolumab,” Oral Oncology 101 (2020): 104525. 10.1016/j.oraloncology.2019.104525 31863963

[43] R. Mehra , T. Y. Seiwert , S. Gupta , et al., “Efficacy and Safety of Pembrolizumab in Recurrent/Metastatic Head and Neck Squamous Cell Carcinoma: Pooled Analyses After Long‐Term Follow‐Up in KEYNOTE‐012,” British Journal of Cancer 119, no. 2 (2018): 153–159. 10.1038/s41416-018-0131-9 PMC6048158 29955135

[44] K. Minohara , T. Matoba , D. Kawakita , et al., “Novel Prognostic Score for Recurrent or Metastatic Head and Neck Cancer Patients Treated With Nivolumab,” Scientific Reports 11, no. 1 (2021): 16992. 10.1038/s41598-021-96538-7 PMC8379150 34417539

[45] T. Okada , C. Fushimi , T. Matsuki , et al., “Effects of Pembrolizumab in Recurrent/Metastatic Squamous Cell Head and Neck Carcinoma: A Multicenter Retrospective Study,” Anticancer Research 43, no. 6 (2023): 2717–2724. 10.21873/anticanres.16438 37247908

[46] I. Okamoto , K. Tsukahara , and H. Sato , “Single‐Center Prospective Study on the Efficacy of Nivolumab Against Platinum‐Sensitive Recurrent or Metastatic Head and Neck Squamous Cell Carcinoma,” Scientific Reports 12, no. 1 (2022): 2025. 10.1038/s41598-022-06084-z PMC8821556 35132165

[47] S. Otsuki , R. Hori , S. Shinohara , et al., “Real‐World 2‐Year Long‐Term Outcomes and Prognostic Factors in Patients Receiving Nivolumab Therapy for Recurrent or Metastatic Squamous Cell Carcinoma of the Head and Neck,” Auris, Nasus, Larynx 49, no. 5 (2022): 834–844. 10.1016/j.anl.2022.02.006 35232636

[48] M. Otsuru , N. Yamakawa , T. Kirita , et al., “Efficacy and Safety of Molecularly Targeted Agents and Immune Checkpoint Inhibitors for Unresectable or Recurrent/Metastatic Oral Cancer in Japan,” Journal of Dental Sciences 19, no. 3 (2024): 1628–1637. 10.1016/j.jds.2023.11.024 PMC11259634 39035308

[49] A. Pareek , A. A. Patel , M. Kumar , et al., “Nivolumab in Recurrent/Metastatic Squamous Cell Carcinoma of Head and Neck: A Tertiary Cancer Center Experience,” South Asian Journal of Cancer 11, no. 1 (2022): 58–61. 10.1055/s-0041-1733317 PMC9273311 35833038

[50] D. Rischin , K. J. Harrington , R. Greil , et al., “Pembrolizumab Alone or With Chemotherapy for Recurrent or Metastatic Head and Neck Squamous Cell Carcinoma: Health‐Related Quality‐of‐Life Results From KEYNOTE‐048,” Oral Oncology 128 (2022): 105815. 10.1016/j.oraloncology.2022.105815 35381576

[51] D. Sano , M. Tokuhisa , H. Takahashi , et al., “Real‐World Therapeutic Outcomes of the Pembrolizumab Regimen as First‐Line Therapy for Recurrent/Metastatic Squamous Cell Carcinoma of the Head and Neck: A Single‐Center Retrospective Cohort Study in Japan,” Anticancer Research 42, no. 9 (2022): 4477–4484. 10.21873/anticanres.15948 36039457

[52] N. H. Segal , S.‐H. I. Ou , A. Balmanoukian , et al., “Safety and Efficacy of Durvalumab in Patients With Head and Neck Squamous Cell Carcinoma: Results From a Phase I/II Expansion Cohort,” European Journal of Cancer (Oxford, England: 1990) 109 (2019): 154–161. 10.1016/j.ejca.2018.12.029 30731276

[53] T. Y. Seiwert , B. Burtness , R. Mehra , et al., “Safety and Clinical Activity of Pembrolizumab for Treatment of Recurrent or Metastatic Squamous Cell Carcinoma of the Head and Neck (KEYNOTE‐012): An Open‐Label, Multicentre, Phase 1b Trial,” Lancet Oncology 17, no. 7 (2016): 956–965. 10.1016/S1470-2045(16)30066-3 27247226

[54] A. Simon , C. Abdeddaim , D. Nguyen , et al., “First‐Line Therapy in Recurrent or Metastatic Head and Neck Squamous Cell Carcinoma: A Retrospective, Multicenter, Real‐World Study,” Head & Neck 47, no. 11 (2025): 2939–2949. 10.1002/hed.28211 PMC12541674 40491285

[55] L. L. Siu , C. Even , R. Mesía , et al., “Safety and Efficacy of Durvalumab With or Without Tremelimumab in Patients With PD‐L1–Low/Negative Recurrent or Metastatic HNSCC: The Phase 2 CONDOR Randomized Clinical Trial,” JAMA Oncology 5, no. 2 (2019): 195–203. 10.1001/jamaoncol.2018.4628 PMC6439564 30383184

[56] M. Tahara , N. Kiyota , K.‐I. Nibu , A. Akamatsu , T. Hoshino , and R. Hayashi , “Real‐World Safety and Effectiveness of Nivolumab for Recurrent or Metastatic Head and Neck Cancer in Japan: A Post‐Marketing Surveillance,” International Journal of Clinical Oncology 26, no. 9 (2021): 1619–1627. 10.1007/s10147-021-01949-1 PMC8364900 34110532

[57] A. Thapa , A. Cowell , A. Peters , et al., “The UK Divide: Does Having a Pembrolizumab–Chemotherapy Option in Head and Neck Cancer Matter? Real‐World Experience of First‐Line Palliative Pembrolizumab Monotherapy and Pembrolizumab–Chemotherapy Combination in Scotland,” Clinical Oncology 36, no. 5 (2024): 287–299. 10.1016/j.clon.2024.02.004 38395634

[58] U. Tsutomu , C. Nobuyuki , T. Masaya , et al., “Baseline Neutrophil‐to‐Lymphocyte Ratio (NLR) is Associated With Clinical Outcome in Recurrent or Metastatic Head and Neck Cancer Patients Treated With Nivolumab,” Acta Oto‐Laryngologica. Supplementum 140, no. 2 (2020): 181–187. 10.1080/00016489.2019.1699250 31825711

[59] Y. Ueki , T. Takahashi , H. Ota , R. Shodo , K. Yamazaki , and A. Horii , “Predicting the Treatment Outcome of Nivolumab in Recurrent or Metastatic Head and Neck Squamous Cell Carcinoma: Prognostic Value of Combined Performance Status and Modified Glasgow Prognostic Score,” European Archives of Oto‐Rhino‐Laryngology 277, no. 8 (2020): 2341–2347. 10.1007/s00405-020-05945-5 32239313

[60] Y. Ueki , S. Ohshima , J. Omata , et al., “Role of Eosinophilia in Patients With Recurrent/Metastatic Head and Neck Squamous Cell Carcinoma Treated With Nivolumab: Prediction of Immune‐Related Adverse Events and Favorable Outcome,” Cancer Medicine 12, no. 22 (2023): 20810–20820. 10.1002/cam4.6648 PMC10709726 37902133

[61] I. Vasiliadou , O. Breik , H. Baker , et al., “Safety and Treatment Outcomes of Nivolumab for the Treatment of Recurrent or Metastatic Head and Neck Squamous Cell Carcinoma: Retrospective Multicenter Cohort Study,” Cancers (Basel) 13, no. 6 (2021): 1–12. 10.3390/cancers13061413 PMC8003537 33808781

[62] I. Vasiliadou , D. Grose , C. Wilson , et al., “The Use of Pembrolizumab Monotherapy for the Management of Head and Neck Squamous Cell Carcinoma (HNSCC) in the UK,” International Journal of Cancer 155, no. 5 (2024): 883–893. 10.1002/ijc.34963 38685816

[63] N. Yamakawa , M. Umeda , Y. Yoshii , et al., “Multicenter Retrospective Study of Nivolumab for Recurrent/Metastatic Oral Squamous Cell Carcinoma,” Oral Diseases 30, no. 2 (2024): 247–258. 10.1111/odi.14471 36519515

[64] R. Yasumatsu , M. Matsuo , T. Wakasaki , et al., “Clinical Outcome in Recurrent and/or Metastatic Head and Neck Cancer Patients After Discontinuation of Nivolumab Monotherapy due to Immune‐Related Adverse Events,” Acta Oto‐Laryngologica 140, no. 12 (2020): 1043–1048. 10.1080/00016489.2020.1807601 32808841

[65] R. Yasumatsu , Y. Shimizu , N. Hanai , et al., “Outcomes of Long‐Term Nivolumab and Subsequent Chemotherapy in Japanese Patients With Head and Neck Cancer: 2‐Year Follow‐Up From a Multicenter Real‐World Study,” International Journal of Clinical Oncology 27, no. 1 (2022): 95–104. 10.1007/s10147-021-02047-y PMC8732924 34773525

[66] C.‐J. Yen , N. Kiyota , N. Hanai , et al., “Two‐Year Follow‐Up of a Randomized Phase III Clinical Trial of Nivolumab vs. the Investigator's Choice of Therapy in the Asian Population for Recurrent or Metastatic Squamous Cell Carcinoma of the Head and Neck (CheckMate 141),” Head & Neck 42, no. 10 (2020): 2852–2862. 10.1002/hed.26331 PMC7540331 32583557

[67] D. P. Zandberg , A. P. Algazi , A. Jimeno , et al., “Durvalumab for Recurrent or Metastatic Head and Neck Squamous Cell Carcinoma: Results From a Single‐Arm, Phase II Study in Patients With >=25% Tumour Cell PD‐L1 Expression Who Have Progressed on Platinum‐Based Chemotherapy,” European Journal of Cancer (Oxford, England: 1990) 107 (2019): 142–152. 10.1016/j.ejca.2018.11.015 30576970

[68] D. I. Rosenthal , T. R. Mendoza , M. S. Chambers , et al., “Measuring Head and Neck Cancer Symptom Burden: The Development and Validation of the M. D. Anderson Symptom Inventory, Head and Neck Module,” Head & Neck 29, no. 10 (2007): 923–931. 10.1002/hed.20602 17358040

[69] A. R. Hansen , K. Ala‐leppilampi , C. McKillop , et al., “Development of the Functional Assessment of Cancer Therapy‐Immune Checkpoint Modulator (FACT‐ICM): A Scale to Measure Quality of Life in Cancer Patients Treated With ICMs,” Annals of Oncology 30 (2019): v519. 10.1002/cncr.32692 31914209

[70] M. A. List , L. L. D'Antonio , D. F. Cella , et al., “The Performance Status Scale for Head and Neck Cancer Patients and the Functional Assessment of Cancer Therapy‐Head and Neck Scale: A Study of Utility and Validity,” Cancer 77, no. 11 (1996): 2294–2301. 10.1002/(SICI)1097-0142(19960601)77:11<2294::AID-CNCR17>3.0.CO;2-S 8635098

[71] K. J. Harrington , R. L. Ferris , M. Gillison , et al., “Efficacy and Safety of Nivolumab Plus Ipilimumab vs Nivolumab Alone for Treatment of Recurrent or Metastatic Squamous Cell Carcinoma of the Head and Neck: The Phase 2 CheckMate 714 Randomized Clinical Trial,” JAMA Oncology 9, no. 6 (2023): 779–789. 10.1001/jamaoncol.2023.0147 PMC10080406 37022706

[72] N. Kiyota , Y. Hasegawa , S. Takahashi , et al., “A Randomized, Open‐Label, Phase III Clinical Trial of Nivolumab vs. Therapy of Investigator's Choice in Recurrent Squamous Cell Carcinoma of the Head and Neck: A Subanalysis of Asian Patients Versus the Global Population in Checkmate 141,” Oral Oncology 73 (2017): 138–146. 10.1016/j.oraloncology.2017.07.023 28939066

[73] X. Li , Q. Fang , W. Du , X. Zhang , L. Dai , and Y. Qiao , “Induction Chemotherapy Combined With Immunotherapy in Locally Advanced Head and Neck Squamous Cell Carcinoma,” BMC Cancer 21, no. 1 (2021): 622. 10.1186/s12885-021-08373-8 PMC8157730 34044810

[74] R. Upadhyay , E. Gogineni , G. Tocaj , et al., “Palliative Quad Shot Radiation Therapy With or Without Concurrent Immune Checkpoint Inhibition for Head and Neck Cancer,” Cancers (Basel) 16, no. 5 (2024): 1049. 10.3390/cancers16051049 PMC10931206 38473406

[75] J.‐P. Machiels , Y. Tao , L. Licitra , et al., “Pembrolizumab Plus Concurrent Chemoradiotherapy Versus Placebo Plus Concurrent Chemoradiotherapy in Patients With Locally Advanced Squamous Cell Carcinoma of the Head and Neck (KEYNOTE‐412): A Randomised, Double‐Blind, Phase 3 Trial,” Lancet Oncology 25, no. 5 (2024): 572–587. 10.1016/S1470-2045(24)00100-1 38561010

[76] V. M. Patil , V. Noronha , N. Menon , et al., “Low‐Dose Immunotherapy in Head and Neck Cancer: A Randomized Study,” Journal of Clinical Oncology 41, no. 2 (2023): 222–232. 10.1200/JCO.22.01015 36265101

[77] R. Haddad , J. Fayette , M. Teixeira , et al., “Atezolizumab in High‐Risk Locally Advanced Squamous Cell Carcinoma of the Head and Neck: A Randomized Clinical Trial,” Journal of the American Medical Association 333, no. 18 (2025): 1599–1607. 10.1001/jama.2025.1483 PMC11907359 40079944

[78] Y. Tao , J. Biau , X. S. Sun , et al., “Pembrolizumab Versus Cetuximab Concurrent With Radiotherapy in Patients With Locally Advanced Squamous Cell Carcinoma of Head and Neck Unfit for Cisplatin (GORTEC 2015‐01 PembroRad): A Multicenter, Randomized, Phase II Trial,” Annals of Oncology 34, no. 1 (2023): 101–110. 10.1016/j.annonc.2022.10.006 36522816

[79] Y. Tao , A. Auperin , X. Sun , et al., “Avelumab‐Cetuximab‐Radiotherapy Versus Standards of Care in Locally Advanced Squamous‐Cell Carcinoma of the Head and Neck: The Safety Phase of a Randomised Phase III Trial GORTEC 2017‐01 (REACH),” European Journal of Cancer (Oxford, England: 1990) 141 (2020): 21–29. 10.1016/j.ejca.2020.09.008 33125944

[80] B. J. Schneider , J. Naidoo , B. D. Santomasso , et al., “Management of Immune‐Related Adverse Events in Patients Treated With Immune Checkpoint Inhibitor Therapy: ASCO Guideline Update,” Journal of Clinical Oncology 39, no. 36 (2021): 4073–4126. 10.1200/JCO.21.01440 34724392

[81] Y.‐D. Yan , Y. Zhao , C. Zhang , et al., “Toxicity Spectrum of Immunotherapy in Advanced Lung Cancer: A Safety Analysis From Clinical Trials and a Pharmacovigilance System,” EClinicalMedicine 50 (2022): 101535. 10.1016/j.eclinm.2022.101535 PMC9256649 35812997

[82] A. Ezdoglian , M. Tsang‐A‐Sjoe , F. Khodadust , et al., “Monocyte‐Related Markers as Predictors of Immune Checkpoint Inhibitor Efficacy and Immune‐Related Adverse Events: A Systematic Review and Meta‐Analysis,” Cancer Metastasis Reviews 44, no. 1 (2025): 35. 10.1007/s10555-025-10246-6 PMC11845441 39982537

[83] J. B. Vermorken , R. Mesia , F. Rivera , et al., “Platinum‐Based Chemotherapy Plus Cetuximab in Head and Neck Cancer,” New England Journal of Medicine 359, no. 11 (2008): 1116–1127. 10.1056/NEJMoa0802656 18784101

[84] T. M. Atkinson , S. J. Ryan , A. V. Bennett , et al., “The Association Between Clinician‐Based Common Terminology Criteria for Adverse Events (CTCAE) and Patient‐Reported Outcomes (PRO): A Systematic Review,” Supportive Care in Cancer 24, no. 8 (2016): 3669–3676. 10.1007/s00520-016-3297-9 PMC4919215 27260018

[85] F. S. de Oliveira , G. Hurwitz , J. Kim , et al., “Identifying Patient‐Reported Outcome Measures (PROMs) for Routine Surveillance of Physical and Emotional Symptoms in Head and Neck Cancer Populations: A Systematic Review,” Journal of Clinical Medicine 10, no. 18 (2021): 4162. 10.3390/jcm10184162 PMC8470145 34575271

[86] J. Haanen , M. Obeid , L. Spain , et al., “Management of Toxicities From Immunotherapy: ESMO Clinical Practice Guideline for Diagnosis, Treatment and Follow‐Up,” Annals of Oncology 33, no. 12 (2022): 1217–1238. 10.1016/j.annonc.2022.10.001 36270461

[87] J. R. Brahmer , C. Lacchetti , B. J. Schneider , et al., “Management of Immune‐Related Adverse Events in Patients Treated With Immune Checkpoint Inhibitor Therapy: American Society of Clinical Oncology Clinical Practice Guideline,” Journal of Clinical Oncology 36, no. 17 (2018): 1714–1768. 10.1200/JCO.2017.77.6385 PMC6481621 29442540

[88] L. R. Wall , E. C. Ward , B. Cartmill , A. J. Hill , and S. V. Porceddu , “Adherence to a Prophylactic Swallowing Therapy Program During (Chemo) Radiotherapy: Impact of Service‐Delivery Model and Patient Factors,” Dysphagia 32, no. 2 (2017): 279–292. 10.1007/s00455-016-9757-z 27844152

[89] O. Strachna , M. A. Cohen , M. M. Allison , et al., “Case Study of the Integration of Electronic Patient‐Reported Outcomes as Standard of Care in a Head and Neck Oncology Practice: Obstacles and Opportunities,” Cancer 127, no. 3 (2021): 359–371. 10.1002/cncr.33272 PMC8375640 33107986

[90] S. Ossowski , A. Kammerer , D. Stram , L. Piazza‐DeLap , E. Basch , and J. A. Katzel , “Patient‐Reported Outcomes Integrated Within an Electronic Medical Record in Patients With Head and Neck Cancer,” JCO Clinical Cancer Informatics 5 (2021): 842–848. 10.1200/CCI.21.00058 34406801

[91] J. Lai‐Kwon , M. Jefford , S. Best , et al., “Mapping Immune Checkpoint Inhibitor Side Effects to Item Libraries for Use in Real‐Time Side Effect Monitoring Systems,” Journal of Patient‐Reported Outcomes 9, no. 1 (2025): 27. 10.1186/s41687-025-00855-8 PMC11885683 40048089

[92] J. Lai‐Kwon , C. Rutherford , S. Best , et al., “Co‐Design of an Electronic Patient‐Reported Outcome Symptom Monitoring System for Immunotherapy Toxicities,” Supportive Care in Cancer 32, no. 12 (2024): 843. 10.1007/s00520-024-09034-9 39623054

[93] S. Nolte , G. Liegl , M. A. Petersen , et al., “General Population Normative Data for the EORTC QLQ‐C30 Health‐Related Quality of Life Questionnaire Based on 15,386 Persons Across 13 European Countries, Canada and the Unites States,” European Journal of Cancer 107 (2019): 153–163. 10.1016/j.ejca.2018.11.024 30576971

[94] I. Brook , “Late Side Effects of Radiation Treatment for Head and Neck Cancer,” Radiation Oncology Journal 38, no. 2 (2020): 84–92. 10.3857/roj.2020.00213 PMC7533405 33012151

[95] K. J. Taylor , C. D. Amdal , K. Bjordal , et al., “Toxicities in Long‐Term Survivors of Head and Neck Cancer—A Multi‐National Cross‐Sectional Analysis,” International Journal of Cancer 157, no. 10 (2025): 2061–2070. 10.1002/ijc.70033 PMC12439067 40735927

[96] E. K. Ruud Kjær , J. S. Jensen , K. K. Jakobsen , et al., “The Impact of Comorbidity on Survival in Patients With Head and Neck Squamous Cell Carcinoma: A Nationwide Case‐Control Study Spanning 35 Years,” Frontiers in Oncology 10 (2021): 617184. 10.3389/fonc.2020.617184 PMC7928275 33680938

[97] A. Winter , S. M. Schulz , M. Schmitter , et al., “Comprehensive Geriatric Assessment and Quality of Life Aspects in Patients With Recurrent/Metastatic Head and Neck Squamous Cell Carcinoma (HNSCC),” Journal of Clinical Medicine 12, no. 17 (2023): 5738. 10.3390/jcm12175738 PMC10488489 37685806

[98] R. Jiang , M. Fritz , and S. K. T. Que , “Cutaneous Squamous Cell Carcinoma: An Updated Review,” Cancers (Basel) 16, no. 10 (2024): 1800. 10.3390/cancers16101800 PMC11119634 38791879

