# Supplementary material for: Treatment‐Related Adverse Events and Health‐Related Quality of Life Associated With Immune Checkpoint Inhibitor Treatment for Mucosal Head and Neck Squamous Cell Carcinoma: A Scoping Review
Source: Head Neck. 2026 Jun 1;48(11):3152–72. doi: 10.1002/hed.70306 (PMC13653308; doi:10.1002/hed.70306)
Supplement: Supplementary file 1 [file HED-48-3152-s001.docx]

# Search Strategies

## MEDLINE

| Set | Search Statement |
| --- | --- |
| 1 | "Head and Neck Neoplasms"/ |
| 2 | ("head and neck cancer" or HNSCC or "head and neck squamous cell carcinoma").ti,ab,kf. |
| 3 | 1 or 2 |
| 4 | Immunotherapy/ |
| 5 | Antineoplastic Agents, Immunological/ |
| 6 | Immune Checkpoint Inhibitors/ |
| 7 | (immunotherap* or "checkpoint inhibitor" or nivolumab or pembrolizumab or PD-1 or PD-L1).ti,ab,kf. |
| 8 | 4 or 5 or 6 or 7 |
| 9 | "Signs and Symptoms"/ |
| 10 | "Drug-Related Side Effects and Adverse Reactions"/ |
| 11 | Symptom Burden/ |
| 12 | "Quality of Life"/ |
| 13 | Needs Assessment/ |
| 14 | Psychological Distress/ |
| 15 | (iRAE or "immune related adverse" or "immune-related adverse" or symptom* or side-effect* or "side effect*" or toxicit* or safety or "supportive care" or "unmet need" or "quality of life").ti,ab,kf. |
| 16 | 9 or 10 or 11 or 12 or 13 or 14 or 15 |
| 17 | 3 and 8 and 16 |

## CINAHL

| Set | Search Statement |
| --- | --- |
| S1 | (MH "Head and Neck Neoplasms+") |
| S2 | TI ("head and neck cancer" OR HNSCC OR "head and neck squamous cell carcinoma") OR AB ("head and neck cancer" OR HNSCC OR "head and neck squamous cell carcinoma") |
| S3 | S1 OR S2 |
| S4 | (MH "Immunotherapy+") |
| S5 | (MH "Antineoplastic Agents, Immunologic+") |
| S6 | TI (immunotherap* OR "checkpoint inhibitor" OR nivolumab OR pembrolizumab OR "pd-1" OR "pd-l1") OR AB (immunotherap* OR "checkpoint inhibitor" OR nivolumab OR pembrolizumab OR "pd-1" OR "pd-l1") |
| S7 | S4 OR S5 OR S6 |
| S8 | (MH "Signs and Symptoms+") |
| S9 | (MH "Drug-Related Side Effects and Adverse Reactions+") |
| S10 | (MH "Quality of Life+") |
| S11 | (MH "Psychological Distress+") |
| S12 | TI (irae OR "immune related adverse" OR "immune-related adverse" OR symptom* OR toxicit* OR "side effect*" OR "side-effect*" OR "quality of life" OR "unmet need" OR "supportive care") OR AB (irae OR "immune related adverse" OR "immune-related adverse" OR symptom* OR toxicit* OR "side effect*" OR "side-effect*" OR "quality of life" OR "unmet need" OR "supportive care") |
| S13 | S8 OR S9 OR S10 OR S11 OR S12 |
| S14 | S3 AND S7 AND S13 |

## Embase

1. 'head and neck tumor'/exp

2. ("head and neck cancer" OR hnscc OR "head and neck squamous cell carcinoma").ti,ab,kw,de

3. 1 OR 2

4. 'immunotherapy'/exp

5. 'antineoplastic immunotherapy agent'/exp

6. 'immune checkpoint inhibitor'/exp

7. (immunotherap* OR "checkpoint inhibitor" OR nivolumab OR pembrolizumab OR "pd-1" OR "pd-l1").ti,ab,kw,de

8. 4 OR 5 OR 6 OR 7

9. 'sign and symptom'/exp

10. 'drug related side effect'/exp

11. 'quality of life'/exp

12. 'psychological stress'/exp

13. (irae OR "immune related adverse" OR "immune-related adverse" OR symptom* OR toxicit* OR "side effect*" OR "side-effect*" OR "quality of life" OR "unmet need" OR "supportive care").ti,ab,kw,de

14. 9 OR 10 OR 11 OR 12 OR 13

15. 3 AND 8 AND 14

## Scopus

- TITLE-ABS-KEY(("head and neck" W/3 cancer OR hnscc OR "head and neck squamous cell carcinoma") AND (immunotherap* OR "checkpoint inhibitor" OR nivolumab OR pembrolizumab OR "pd-1" OR "pd-l1") AND (irae OR "immune related adverse" OR "immune-related adverse" OR symptom* OR toxicit* OR "side effect*" OR "side-effect*" OR "quality of life" OR "unmet need" OR "supportive care"))
