# Supplementary material for: Treatment‐Related Adverse Events and Health‐Related Quality of Life Associated With Immune Checkpoint Inhibitor Treatment for Mucosal Head and Neck Squamous Cell Carcinoma: A Scoping Review
Source: Head Neck. 2026 Jun 1;48(11):3152–72. doi: 10.1002/hed.70306 (PMC13653308; doi:10.1002/hed.70306)
Supplement: Supplementary file 2 [file HED-48-3152-s002.docx]

*Supplementary Table 1. Combined treatment in R/M HNSCC*

| Study | Country | Design | ICI agent(s) | Combination regimen(s) |
| --- | --- | --- | --- | --- |
| Altay-Langguth et al. ^1^ | Germany | Retrospective cohort study | Nivolumab | XRT + ICI |
| Cabezas-Camarero et al. ^2^ | Spain | Retrospective cohort study | Pembrolizumab | chemotherapy + ICI |
| Chung et al. ^3^ | United States | Non randomized phase 1/2 trial | Nivolumab | cetuximab + ICI |
| Dzienis et al. ^4^ | International | Non randomized phase 4 trial | Pembrolizumab | chemotherapy + ICI |
| Fuereder et al. ^5^ | Austria | Non randomized phase 1/2 trial | Pembrolizumab | chemotherapy + ICI |
| Fan et al. ^6^ | China | Retrospective cohort study | Pembrolizumab | Cohort 1: chemotherapy + ICI  Cohort 2: cetuximab + ICI |
| Gui et al. ^7^ | China | Non randomized phase 2 trial | Pembrolizumab | chemotherapy + ICI |
| Gulati et al. ^8^ | United States | Non randomized phase 2 trial | Durvalumab | cetuximab + ICI |
| Hu et al. ^9^ | China | Retrospective cohort study | Pembrolizumab | chemotherapy + ICI; cetuximab + ICI; XRT + ICI; ICI monotherapy (results pooled across regimens) |
| Hui et al. ^10^ | China | Retrospective cohort study | Non specified ICI | cetuximab + ICI |
| Khadela et al. ^11^ | India | Randomized phase 4 trial | Nivolumab | chemotherapy + ICI |
| Koukourakis et al. ^12^ | Greece | Retrospective cohort study | Nivolumab | XRT + ICI |
| Lee et al. ^13^ | United States | Retrospective cohort study | Pembrolizumab | chemotherapy + ICI |
| Li et al. ^14^ | China | Non randomized clinical trial | Pembrolizumab  Toripalimab  Camrelizumab | chemotherapy + XRT + ICI |
| Machiels et al. ^15^ | International | Randomized phase 3 trial | Pembrolizumab | chemotherapy + XRT + ICI |
| Madabhavi et al. ^16^ | India | Prospective cohort study | Nivolumab | chemotherapy + ICI |
| Matsuki et al. ^17^ | Japan | Retrospective cohort study | Pembrolizumab | chemotherapy + ICI; ICI monotherapy (results pooled across regimens) |
| Mell et al. ^18^ | United States | Randomized phase 2/3 trial | Durvalumab | XRT + ICI |
| Merlano et al. ^19^ | Italy | Non randomized phase 1/2 trial | Avelumab | chemotherapy + XRT + ICI |
| Miyamaru et al. ^20^ | Japan | Retrospective cohort study | Pembrolizumab | chemotherapy + ICI; ICI monotherapy (results pooled across regimens) |
| Patil et al. ^21^ | India | Randomized phase 3 trial | Nivolumab | chemotherapy + ICI |
| Saba et al. ^22^ | United States | Non randomized phase 2 trial | Nivolumab | XRT + ICI |
| Sacco et al. ^23^ | United States | Non randomized phase 2 trial | Pembrolizumab | cetuximab + ICI |
| Upadhyay et al. ^24^ | United States | Retrospective cohort | Pembrolizumab  Nivolumab | XRT + ICI |

Abbreviations: ICI, immune checkpoint inhibitor; XRT, radiation therapy

*Supplementary Table 2. Combined treatment in LA HNSCC*

| Study | Country | Design | ICI agent(s) | Timing of ICI administration | Combination regimen(s) |
| --- | --- | --- | --- | --- | --- |
| Bonomo et al. ^25^ | Italy | Non randomized phase 1/2 trial | Durvalumab | Concurrent and maintenance | XRT + cetuximab + ICI followed by maintenance ICI |
| Elbers et al. ^26^ | The Netherlands | Non randomized phase 1 trial | Avelumab | Concurrent and maintenance | XRT + cetuximab + ICI followed by maintenance ICI |
| Ferris et al. (2021) | International | Non randomized phase 1/2 trial | Nivolumab | Neoadjuvant | ICI neoadjuvant to surgery |
| Gillison et al. ^27^ | United States | Non randomized clinical trial | Nivolumab | Concurrent | Arm 1, 2: ICI + chemotherapy + XRT  Arm 3: ICI + cetuximab + XRT  Arm 4: ICI + XRT |
| Haddad et al. ^28^ | International | Randomized phase 3 trial | Atezolizumab | Maintenance | Arm 1: ICI maintenance post multimodal definitive treatment  Arm 2: placebo maintenance post multimodal definitive treatment |
| Huang et al. ^29^ | China | Non randomized phase 1 trial | Toripalimab | Neoadjuvant (concurrent) | chemotherapy + ICI neoadjuvant to surgery |
| Huang et al. ^30^ | China | Non randomized clinical trial | Toripalimab | Neoadjuvant (concurrent) | chemotherapy + ICI neoadjuvant to surgery |
| Knochelmann et al. ^31^ | United States | Non randomized phase 2 trial | Nivolumab | Neoadjuvant | ICI neoadjuvant to surgery |
| Leidner et al. ^32^ | United States | Non randomized phase 1 trial | Nivolumab | Neoadjuvant (concurrent) | XRT + ICI neoadjuvant to surgery |
| Li et al. ^33^ | China | Non randomized clinical trial | Sintilimab | Neoadjuvant | ICI neoadjuvant to definitive multimodal treatment |
| Machiels et al. ^34^ | International | Randomized phase 3 trial | Pembrolizumab | Concurrent | Arm 1: ICI + chemotherapy + XRT  Arm 2: placebo + chemotherapy + XRT |
| Ou et al. ^35^ | China | Non randomized phase 2 trial | Toripalimab | Neoadjuvant (concurrent) | chemotherapy + ICI neoadjuvant to definitive multimodal treatment |
| Patel et al. ^36^ | United States | Non randomized phase 2 trial | Durvalumab | Neoadjuvant (concurrent) | chemotherapy + ICI neoadjuvant to definitive multimodal treatment |
| Powell et al. ^37^ | United States | Non randomized phase 1 trial | Pembrolizumab | Concurrent | chemotherapy + XRT + ICI |
| Rosenberg et al. ^38^ | United States | Non randomized phase 2 trial | Nivolumab | Neoadjuvant (concurrent) | chemotherapy + ICI neoadjuvant to definitive multimodal treatment |
| Shen et al. ^39^ | China | Retrospective cohort | Nivolumab | Neoadjuvant (concurrent) | XRT + ICI neoadjuvant to surgery |
| Tao et al. ^40^ | Europe | Randomized phase 3 trial | Avelumab | Concurrent | Arm 1: XRT + chemotherapy  Arm 2: XRT + cetuximab  Arm 3: XRT + cetuximab + ICI |
| Tao et al. ^41^ | Europe | Randomized phase 2 trial | Pembrolizumab | Concurrent | Arm 1: XRT + cetuximab  Arm 2: XRT + ICI |
| Uppaluri et al. ^42^ | United States | Non randomized phase 2 trial | Pembrolizumab | Neoadjuvant | ICI neoadjuvant to multimodal definitive treatment |
| Uppaluri et al. ^43^ | United States | Randomized phase 3 trial | Pembrolizumab | Neoadjuvant, concurrent and maintenance | ICI neoadjuvant to multimodal definitive treatment, followed by maintenance ICI |
| Vos et al. ^44^ | The Netherlands | Non randomized phase 1/2 trial | Nivolumab | Neoadjuvant (concurrent) | Arm 1: ICI neoadjuvant to surgery  Arm 2: combination ICI neoadjuvant to surgery |
| Wang et al. ^45^ | China | Non randomized clinical trial | Pembrolizumab | Neoadjuvant (concurrent) | chemotherapy + ICI neoadjuvant to multimodal definitive treatment |
| Weiss et al. ^46^ | United States | Non randomized phase 2 trial | Pembrolizumab | Concurrent | XRT + ICI |
| Wise-Draper et al. ^47^ | United States | Non randomized phase 2 trial | Pembrolizumab | Neoadjuvant, concurrent and maintenance | ICI neoadjuvant to multimodal definitive therapy, followed by maintenance ICI |
| Wu et al. ^48^ | China | Non randomized phase 2 trial | Camrelizumab | Neoadjuvant (concurrent) | chemotherapy + ICI neoadjuvant to multimodal definitive therapy |
| Yao et al. ^49^ | China | Retrospective cohort study | Pembrolizumab | Neoadjuvant (concurrent) | chemotherapy + cetuximab + ICI neoadjuvant to surgery |
| Zhang et al. ^50^ | China | Non randomized phase 2 trial | Camrelizumab | Neoadjuvant (concurrent) | chemotherapy + ICI neoadjuvant to multimodal definitive therapy |
| Zhao et al. ^51^ | China | Non randomized phase 2 trial | Camrelizumab | Neoadjuvant (concurrent) | chemotherapy + ICI neoadjuvant to multimodal definitive therapy |

Abbreviations: ICI, immune checkpoint inhibitor; XRT, radiation therapy

**References**

1.	Altay-Langguth A, Balermpas P, Brandts C, Balster S, Ghanaati S, Winkelmann R, et al. Re-irradiation with concurrent and maintenance nivolumab in locally recurrent and inoperable squamous cell carcinoma of the head and neck: A single-center cohort study. Clinical and Translational Radiation Oncology. 2021;28:71-8.

2.	Cabezas-Camarero S, Iglesias-Moreno MC, Cerezo Druet E, Sotelo MJ, Merino-Menéndez S, Cabrera-Martín MN, et al. Durable complete remission with local therapies after neoadjuvant and adjuvant nivolumab in recurrent/metastatic head and neck cancer. Anticancer Drugs. 2023;34(5):695-8.

3.	Chung CH, Bonomi M, Steuer CE, Li J, Bhateja P, Johnson M, et al. Concurrent cetuximab and nivolumab as a second-line or beyond treatment of patients with recurrent and/or metastatic head and neck squamous cell carcinoma: Results of phase i/ii study. Cancers (Basel). 2021;13(5):1-16.

4.	Dzienis M, Cundom J, Fuentes CS, Spreafico A, Nordlinger M, Pastor AV, et al. Pembrolizumab Plus Carboplatin and Paclitaxel as First-Line Therapy for Recurrent/Metastatic Head and Neck Squamous Cell Carcinoma (KEYNOTE-B10): A Single-Arm Phase IV Trial. Journal of clinical oncology : official journal of the American Society of Clinical Oncology. 2024;42(25):2989-99.

5.	Fuereder T, Minichsdorfer C, Mittlboeck M, Wagner C, Heller G, Putz EM, et al. Pembrolizumab plus docetaxel for the treatment of recurrent/metastatic head and neck cancer: A prospective phase I/II study. Oral Oncol. 2022;124.

6.	Fan Z, Hui R, Ju H, Wu Y, Ma X, Song H, et al. The clinical outcome of pembrolizumab for patients with recurrent or metastatic squamous cell carcinoma of the head and neck: a single center, real world study in China. Front Oncol. 2024;14.

7.	Gui L, Chen X, Zhang W, Xie Z, Zhang Y, Li W, et al. Efficacy, safety, and multi-omics analysis of pembrolizumab combined with nab-paclitaxel and platinum as first-line treatment in patients with recurrent or metastatic head and neck squamous cell carcinoma: A single-arm phase 2 study. Chinese journal of cancer research = Chung-kuo yen cheng yen chiu. 2024;36(6):713-28.

8.	Gulati S, Crist M, Riaz MK, Takiar V, Lehn M, Monroe I, et al. Durvalumab plus Cetuximab in Patients with Recurrent or Metastatic Head and Neck Squamous Cell Carcinoma: An Open-label, Nonrandomized, Phase II Clinical Trial. Clinical cancer research : an official journal of the American Association for Cancer Research. 2023;29(10):1906-15.

9.	Hu M, Li X, Feng H, Qiao Q, Hu G, Yang K, et al. A multicenter, real-world study analysis: pembrolizumab changes treatment strategy for the patient with recurrent/metastatic head and neck squamous cell carcinoma. Cancer Lett. 2025;634:217996.

10.	Hui R, Liu X, Fan Z, Ji H, Wei D, Ren G. The efficacy of cetuximab plus PD-1 inhibitors as salvage therapy in PD-1 refractory patients with recurrent or metastatic head and neck squamous cell carcinoma. J Cancer. 2024;15(6):1668-74.

11.	Khadela A, Parikh R, Vaghela J, Chauhan Y, Shah VB, Kothari R, et al. A Real-World Phase IV Superiority Trial and Cost-Effectiveness Analysis of the Addition of Low-Dose Nivolumab to Triple Metronomic Chemotherapy Compared to the Paclitaxel Carboplatin Regimen in Recurrent or Metastatic Head and Neck Cancer Patients. Head Neck. 2025.

12.	Koukourakis IM, Giakzidis AG, Koukourakis MI. Anti-PD-1 immunotherapy with dose-adjusted ultra-hypofractionated re-irradiation in patients with locoregionally recurrent head and neck cancer. Clinical and Translational Oncology. 2023;25(10):3032-41.

13.	Lee B, Chehab SS, Fan W, Schell MJ, Kirtane KS, Shah AB. Safety outcomes of pembrolizumab with platinum agent chemotherapy combined with 5-fluorouracil or taxane derivative in head and neck cancer. Journal of oncology pharmacy practice : official publication of the International Society of Oncology Pharmacy Practitioners. 2024;30(8):1397-402.

14.	Li L, Chen L, Yan L, Guo Y, Li F, Fan M, et al. Initial analysis of the synergy of programmed cell death-1 (PD-1) inhibitor and concurrent chemoradiotherapy treatment for recurrent/metastatic head and neck squamous cell carcinoma patients. Radiat Oncol. 2023;18(1):109.

15.	Machiels JP, Tao Y, Licitra L, Burtness B, Tahara M, Rischin D, et al. Health-related quality of life outcomes from KEYNOTE-412: chemoradiotherapy with or without pembrolizumab in participants with head and neck squamous cell carcinoma. Front Oncol. 2025;15:1645509.

16.	Madabhavi I, Sarkar M, Kumar V, Sagar R. Combined Metronomic Chemo-immunotherapy (CMCI) in Head and Neck Cancers-An Experience from a Developing Country. Indian journal of surgical oncology. 2024;15(2):321-31.

17.	Matsuki T, Okada T, Fushimi C, Takahashi H, Okamoto I, Kondo T, et al. Hematological Markers Predict Immune-Related Adverse Events in Patients With Recurrent or Metastatic Squamous Cell Carcinoma of the Head and Neck Treated With Pembrolizumab. Asia Pac J Clin Oncol. 2025.

18.	Mell LK, Torres-Saavedra PA, Wong SJ, Kish JA, Chang SS, Jordan RC, et al. Radiotherapy with cetuximab or durvalumab for locoregionally advanced head and neck cancer in patients with a contraindication to cisplatin (NRG-HN004): an open-label, multicentre, parallel-group, randomised, phase 2/3 trial. The Lancet Oncology. 2024;25(12):1576-88.

19.	Merlano MC, Denaro N, Paccagnella M, Abbona A, Galizia D, Alfieri S, et al. Multimodality immunotherapy with avelumab, short-course radiotherapy, and cyclophosphamide in head and neck cancer: the CONFRONT phase I-II trial. ESMO open. 2025;10(4):104498.

20.	Miyamaru S, Murakami D, Nishimoto K, Orita Y. Identifying factors for pembrolizumab eligibility in head and neck cancer. J Cancer Res Clin Oncol. 2025;151(2):67.

21.	Patil VM, Noronha V, Menon N, Rai R, Bhattacharjee A, Singh A, et al. Low-Dose Immunotherapy in Head and Neck Cancer: A Randomized Study. J Clin Oncol. 2023;41(2):222-32.

22.	Saba NF, Wong SJ, Nasti T, McCook-Veal AA, McDonald MW, Stokes WA, et al. Intensity-Modulated Reirradiation Therapy with Nivolumab in Recurrent or Second Primary Head and Neck Squamous Cell Carcinoma: A Nonrandomized Controlled Trial. JAMA Oncology. 2024.

23.	Sacco AG, Chen R, Worden FP, Wong DJL, Adkins D, Swiecicki P, et al. Pembrolizumab plus cetuximab in patients with recurrent or metastatic head and neck squamous cell carcinoma: an open-label, multi-arm, non-randomised, multicentre, phase 2 trial. The Lancet Oncology. 2021;22(6):883-92.

24.	Upadhyay R, Gogineni E, Tocaj G, Ma SJ, Bonomi M, Bhateja P, et al. Palliative Quad Shot Radiation Therapy with or without Concurrent Immune Checkpoint Inhibition for Head and Neck Cancer. Cancers (Basel). 2024;16(5).

25.	Bonomo P, Desideri I, Mangoni M, Saieva C, Loi M, Becherini C, et al. Durvalumab with cetuximab and radiotherapy for locally advanced squamous cell carcinoma of the head and neck: A phase 1/2 trial. Radiotherapy and oncology : journal of the European Society for Therapeutic Radiology and Oncology. 2022;169:64-70.

26.	Elbers JBW, Al-Mamgani A, Tesseslaar MET, van den Brekel MWM, Lange CAH, van der Wal JE, et al. Immuno-radiotherapy with cetuximab and avelumab for advanced stage head and neck squamous cell carcinoma: Results from a phase-I trial. Radiotherapy and oncology : journal of the European Society for Therapeutic Radiology and Oncology. 2020;142:79-84.

27.	Gillison ML, Ferris RL, Harris J, Colevas AD, Mell LK, Kong C, et al. Safety of Nivolumab Added to Chemoradiation Therapy Platforms for Intermediate and High-Risk Locoregionally Advanced Head and Neck Squamous Cell Carcinoma: Radiation Therapy Oncology Group Foundation 3504. Int J Radiat Oncol Biol Phys. 2023;115(4):847-60.

28.	Haddad R, Fayette J, Teixeira M, Prabhash K, Mesia R, Kawecki A, et al. Atezolizumab in High-Risk Locally Advanced Squamous Cell Carcinoma of the Head and Neck: A Randomized Clinical Trial. JAMA. 2025;333(18):1599-607.

29.	Huang X, Liu Q, Zhong G, Peng Y, Liu Y, Liang L, et al. Neoadjuvant toripalimab combined with gemcitabine and cisplatin in resectable locally advanced head and neck squamous cell carcinoma (NeoTGP01): An open label, single-arm, phase Ib clinical trial. Journal of experimental & clinical cancer research : CR. 2022;41(1):300.

30.	Huang Y, Sun J, Li J, Zhu D, Dong M, Dou S, et al. Neoadjuvant immunochemotherapy for locally advanced resectable oral squamous cell carcinoma: a prospective single-arm trial (Illuminate Trial). Int J Surg. 2023;109(8):2220-7.

31.	Knochelmann HM, Horton JD, Liu S, Armeson K, Kaczmar JM, Wyatt MM, et al. Neoadjuvant presurgical PD-1 inhibition in oral cavity squamous cell carcinoma. Cell reports Medicine. 2021;2(10):100426.

32.	Leidner R, Crittenden M, Young K, Xiao H, Wu Y, Couey MA, et al. Neoadjuvant immunoradiotherapy results in high rate of complete pathological response and clinical to pathological downstaging in locally advanced head and neck squamous cell carcinoma. Journal for immunotherapy of cancer. 2021;9(5).

33.	Li X, Fang Q, Du W, Zhang X, Dai L, Qiao Y. Induction chemotherapy combined with immunotherapy in locally advanced head and neck squamous cell carcinoma. BMC Cancer. 2021;21(1).

34.	Machiels J-P, Tao Y, Licitra L, Burtness B, Tahara M, Rischin D, et al. Pembrolizumab plus concurrent chemoradiotherapy versus placebo plus concurrent chemoradiotherapy in patients with locally advanced squamous cell carcinoma of the head and neck (KEYNOTE-412): a randomised, double-blind, phase 3 trial. The Lancet Oncology. 2024;25(5):572-87.

35.	Ou X, Zhai R, Wei W, Chen J, Ou D, Liao T, et al. Induction Toripalimab and Chemotherapy for Organ Preservation in Locally Advanced Laryngeal and Hypopharyngeal Cancer: A Single-Arm Phase II Clinical Trial. Clinical cancer research : an official journal of the American Association for Cancer Research. 2024;30(2):344-55.

36.	Patel SA, Gibson MK, Deal A, Sheth S, Heiling H, Johnson SM, et al. A phase 2 study of neoadjuvant chemotherapy plus durvalumab in resectable locally advanced head and neck squamous cell carcinoma. Cancer. 2023;129(21):3381-9.

37.	Powell SF, Gold KA, Gitau MM, Sumey CJ, Lohr MM, McGraw SC, et al. Safety and Efficacy of Pembrolizumab With Chemoradiotherapy in Locally Advanced Head and Neck Squamous Cell Carcinoma: A Phase IB Study. Journal of clinical oncology : official journal of the American Society of Clinical Oncology. 2020;38(21):2427-37.

38.	Rosenberg AJ, Agrawal N, Juloori A, Cursio J, Gooi Z, Blair E, et al. Neoadjuvant Nivolumab Plus Chemotherapy Followed By Response-Adaptive Therapy for HPV+ Oropharyngeal Cancer: OPTIMA II Phase 2 Open-Label Nonrandomized Controlled Trial. JAMA oncology. 2024;10(7):923-31.

39.	Shen P, Qiao B, Jin N, Wang S. Neoadjuvant immunoradiotherapy in patients with locally advanced oral cavity squamous cell carcinoma: a retrospective study. Invest New Drugs. 2022;40(6):1282-9.

40.	Tao Y, Auperin A, Sun X, Sire C, Martin L, Coutte A, et al. Avelumab-cetuximab-radiotherapy versus standards of care in locally advanced squamous-cell carcinoma of the head and neck: The safety phase of a randomised phase III trial GORTEC 2017-01 (REACH). European journal of cancer (Oxford, England : 1990). 2020;141:21-9.

41.	Tao Y, Biau J, Sun XS, Sire C, Martin L, Alfonsi M, et al. Pembrolizumab versus cetuximab concurrent with radiotherapy in patients with locally advanced squamous cell carcinoma of head and neck unfit for cisplatin (GORTEC 2015-01 PembroRad): a multicenter, randomized, phase II trial. Ann Oncol. 2023;34(1):101-10.

42.	Uppaluri R, Campbell KM, Egloff AM, Zolkind P, Skidmore ZL, Nussenbaum B, et al. Neoadjuvant and Adjuvant Pembrolizumab in Resectable Locally Advanced, Human Papillomavirus-Unrelated Head and Neck Cancer: A Multicenter, Phase II Trial. Clinical cancer research : an official journal of the American Association for Cancer Research. 2020;26(19):5140-52.

43.	Uppaluri R, Haddad Robert I, Tao Y, Le Tourneau C, Lee Nancy Y, Westra W, et al. Neoadjuvant and Adjuvant Pembrolizumab in Locally Advanced Head and Neck Cancer. N Engl J Med. 2025;393(1):37-50.

44.	Vos JL, Elbers JBW, Krijgsman O, Traets JJH, Qiao X, van der Leun AM, et al. Neoadjuvant immunotherapy with nivolumab and ipilimumab induces major pathological responses in patients with head and neck squamous cell carcinoma. Nature Communications. 2021;12(1).

45.	Wang K, Gui L, Lu H, He X, Li D, Liu C, et al. Efficacy and safety of pembrolizumab with preoperative neoadjuvant chemotherapy in patients with resectable locally advanced head and neck squamous cell carcinomas. Front Immunol. 2023;14:1189752.

46.	Weiss J, Sheth S, Deal AM, Grilley Olson JE, Patel S, Hackman TG, et al. Concurrent Definitive Immunoradiotherapy for Patients with Stage III–IV Head and Neck Cancer and Cisplatin Contraindication. Clin Cancer Res. 2020;26(16):4260-7.

47.	Wise-Draper TM, Gulati S, Palackdharry S, Hinrichs BH, Worden FP, Old MO, et al. Phase II Clinical Trial of Neoadjuvant and Adjuvant Pembrolizumab in Resectable Local-Regionally Advanced Head and Neck Squamous Cell Carcinoma. Clinical cancer research : an official journal of the American Association for Cancer Research. 2022;28(7):1345-52.

48.	Wu D, Li Y, Xu P, Fang Q, Cao F, Lin H, et al. Neoadjuvant chemo-immunotherapy with camrelizumab plus nab-paclitaxel and cisplatin in resectable locally advanced squamous cell carcinoma of the head and neck: a pilot phase II trial. Nature Communications. 2024;15(1).

49.	Yao Z, Wang J, Jiang Y, Zhang Y, Liu J, Dai L, et al. Pembrolizumab plus cetuximab with neoadjuvant chemotherapy for head and neck squamous cell carcinoma. Head Neck. 2025;47(1):289-99.

50.	Zhang Z, Wu B, Peng G, Xiao G, Huang J, Ding Q, et al. Neoadjuvant Chemoimmunotherapy for the Treatment of Locally Advanced Head and Neck Squamous Cell Carcinoma: A Single-Arm Phase 2 Clinical Trial. Clin Cancer Res. 2022;28(15):3268-76.

51.	Zhao X, Zhou Y, Peng G, Wen L, Hong X, Hu Y, et al. Final analysis of a phase II trial of neoadjuvant chemoimmunotherapy for locoregionally advanced head and neck squamous cell carcinoma. Oral Oncol. 2024;156:106918.
